# Spatiotemporal patterning of acoustic gaze in echolocating bats navigating gaps in clutter

**DOI:** 10.1016/j.isci.2021.102353

**Published:** 2021-03-23

**Authors:** Amaro Tuninetti, Chen Ming, Kelsey N. Hom, James A. Simmons, Andrea Megela Simmons

**Affiliations:** 1 Department of Cognitive, Linguistic, & Psychological Sciences, Brown University, Providence, RI 02912, USA; 2 Department of Neuroscience, Brown University, Providence, RI 02912, USA; 3 Carney Institute for Brain Science, Brown University, Providence, RI 02912, USA

**Keywords:** Biological Sciences, Zoology, Animals, Ethology

## Abstract

We challenged four big brown bats to maneuver through abrupt turns in narrow corridors surrounded by dense acoustic clutter. We quantified bats' performance, sonar beam focus, and sensory acquisition rate. Performance was excellent in straight corridors, with sonar beam aim deviating less than 5° from the corridor midline. Bats anticipated an upcoming abrupt turn to the right or left by slowing flight speed and shifting beam aim to “look” proactively into one side of the corridor to identify the new flightpath. All bats mastered the right turn, but two bats consistently failed the left turn. Bats increased their sensory acquisition rate when confronting abrupt turns in both successful and failed flights. Limitations on biosonar performance reflected failures to switch beam aim and to modify a learned spatial map, rather than failures to update acquisition rate.

## Highlights

• Echolocating bats use sonar to identify flightpaths through dense, cluttered scenes
• Bats learn new flightpaths by switching beam aim before encountering obstacles
• Bats see their environment in depth

## Graphical abstract

**Figure:**
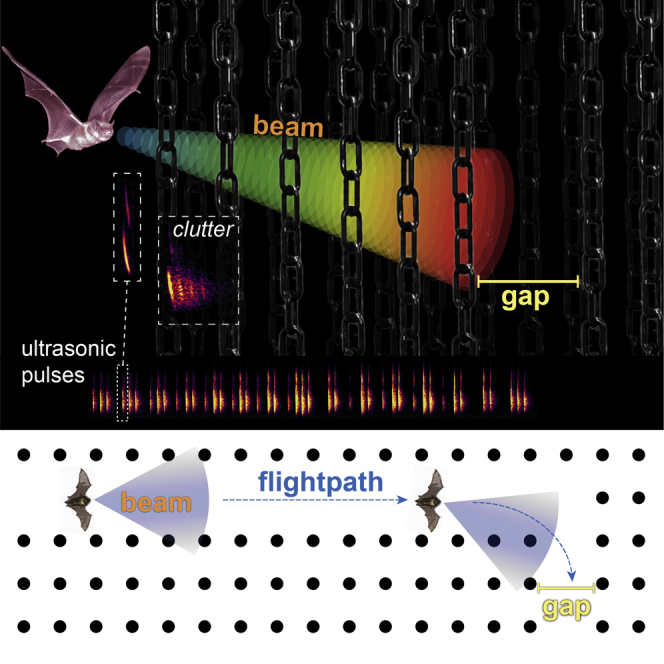

## Introduction

Echolocating bats intercept flying prey and navigate through their environments using information provided by reflected echoes. To identify obstacles and open flightpaths, they actively modulate the number, temporal patterning, and spatial direction of their biosonar calls (Griffin, 1958; Moss and Surlykke, 2001; Surlykke and Moss, 2000). Many bat species forage and navigate in noisy, highly echoic (cluttered) environments, such as vegetation, forests, and caves (Schnitzler and Kalko, 2001), that challenge their biosonar system to decipher multiple, dynamic streams of echoes from numerous objects located at different distances. In these noisy environments, bats maintain flightpaths, detect specific (prey) objects in surrounding (non-prey) background clutter, identify particular echoes in multiple overlapping streams of echoes, and selectively attend to relevant echoes amid a stream of irrelevant ones that constitute the clutter (Amichai and Yovel, 2017; Beetz et al., 2019; Fujioka et al., 2014; Hiryu et al., 2010; Knowles et al., 2015; Melcón et al., 2011; Petrites et al., 2009; Sändig et al., 2014; Surlykke et al., 2009; Wheeler et al., 2016). The experiments described here analyze how one species of FM (frequency-modulated) bat, the big brown bat, *Eptesicus fuscus*, modifies the direction and timing of its biosonar calls when flying through a cluttered environment requiring maneuvers through tight turns. As in natural clutter, multiple objects in this environment are located nearer than the upcoming opening in the scene, so the bat has to peer through a screen of echoes to discern the place where a turn will be necessary to maintain the flightpath.

Big brown bats have a versatile biosonar system. They capture prey in open spaces as well as on the ground and in or near vegetation; they fly above the forest canopy, between gaps in foliage, and within caves and other roosting spots (Simmons, 2005; Simmons et al., 2001). Their sonar beam is wide (−6 dB beam width = 55°) and ensonifies a large acoustic field of view (Ghose and Moss, 2003; Hartley and Suthers, 1989). Because wide beamwidths result in echoes returning from all objects within the ensonified field of view, identifying target echoes and then developing a flightpath to maneuver around obstacles or capture a prey item within foliage is not a simple task. Echoes arriving from objects located near the periphery of the sonar beam can mask echoes from objects located near the front (Bates et al., 2011). To contend with this masking problem, big brown bats flying toward a target in an open room sequentially scan their surroundings to center the sonar beam on the target; they then lock onto the target several hundreds of milliseconds before physically approaching it (Surlykke et al., 2009). When navigating down straight corridors surrounded by dense acoustic clutter, they align the center of the beam with the direction of flight, with minimal side-to-side scanning throughout the duration of the flight (Knowles et al., 2015; Warnecke et al., 2016, 2018). It is not known how bats modify the direction of beam aim and their scanning behavior in highly cluttered environments requiring complex flightpaths. Understanding how bats make these modifications in different environments can elucidate the strategies they use to choose open flightpaths and to reject surrounding clutter echoes, and when these strategies might fail (Kounitsky et al., 2015; Grief et al., 2017).

Besides changing beam aim, big brown bats also modify their sensory acquisition rate when flying in cluttered environments compared to flying in the open (Moss et al., 2006; Surlykke and Moss, 2000). In the presence of clutter, bats increase their rate of call (pulse) emission to update their view of the acoustic scene. However, at increased pulse rates, the bat emits a new pulse before all the echoes created by the previous pulse have returned. This overlap in emitted pulses and returning echoes produces pulse-echo ambiguity, the problem in determining which echo corresponds to which emitted pulse and thus in calculating the distance to the specific object that created that echo (Denny, 2007). One way of solving this ambiguity problem is to emit pulses in sonar sound groups (SSGs) of alternating short and long intervals between individual pulses or groups of pulses (Hiryu et al., 2010; Kothari et al., 2014; Moss et al., 2006). Indeed, in the corridor experiments described above, big brown bats emitted more grouped pulses when flying through narrower and more cluttered corridors (Accomando et al., 2018; Petrites et al., 2009; Wheeler et al., 2016). These behaviors are consistent with the hypotheses that changes in pulse timing occur when the bat needs to gather more frequent updates of the acoustic scene (Moss et al., 2006; Falk et al., 2014), and that the presence of SSGs provides an index of the perceptual difficulty of the task to the bat (Kothari et al., 2014; Lewanzik and Goerlitz, 2021; Petrites et al., 2009; Simmons et al., 2020).

One issue with the hypothesis that changes in pulse timing reflect the bat's own interpretation of an acoustic scene as “easy” or “difficult” is that in many flight and obstacle avoidance experiments, big brown bats exhibit excellent performance, with few or no errors (Hom et al., 2016; Knowles et al., 2015; Petrites et al., 2009; Simmons et al., 2018; Wheeler et al., 2016; but see Surlykke et al., 2009). They navigate successfully down straight and curved corridors, as narrow as 40 cm (their wingspan is only 32–35 cm), surrounded by arrays of hanging plastic chains producing dense, extended streams of echoes (Accomando et al., 2018; Petrites et al., 2009; Wheeler et al., 2016), even after exposure to intense levels of noise that would be expected to alter their sensitivity to echoes (Hom et al., 2016; Simmons et al., 2018). Here, we designed a flight task where big brown bats were required to maneuver down a narrow corridor surrounded by acoustic clutter produced by reflective chains and then through abrupt 90° turns. This experimental paradigm broadly mimics an acoustic scene these animals would experience when attempting to locate and fly through gaps between foliage. We made three predictions. First, the inclusion of abrupt turns makes the task challenging for bats to master. Second, bats alter the direction of their beam aim to ensonify the area surrounding the turns well before physically reaching them, and these changes in beam aim are related to performance. Finally, bats call at high rates with pronounced pulse grouping as they approach the turn, and these changes in pulse grouping are related to performance.

## Results

**Figure 1:**
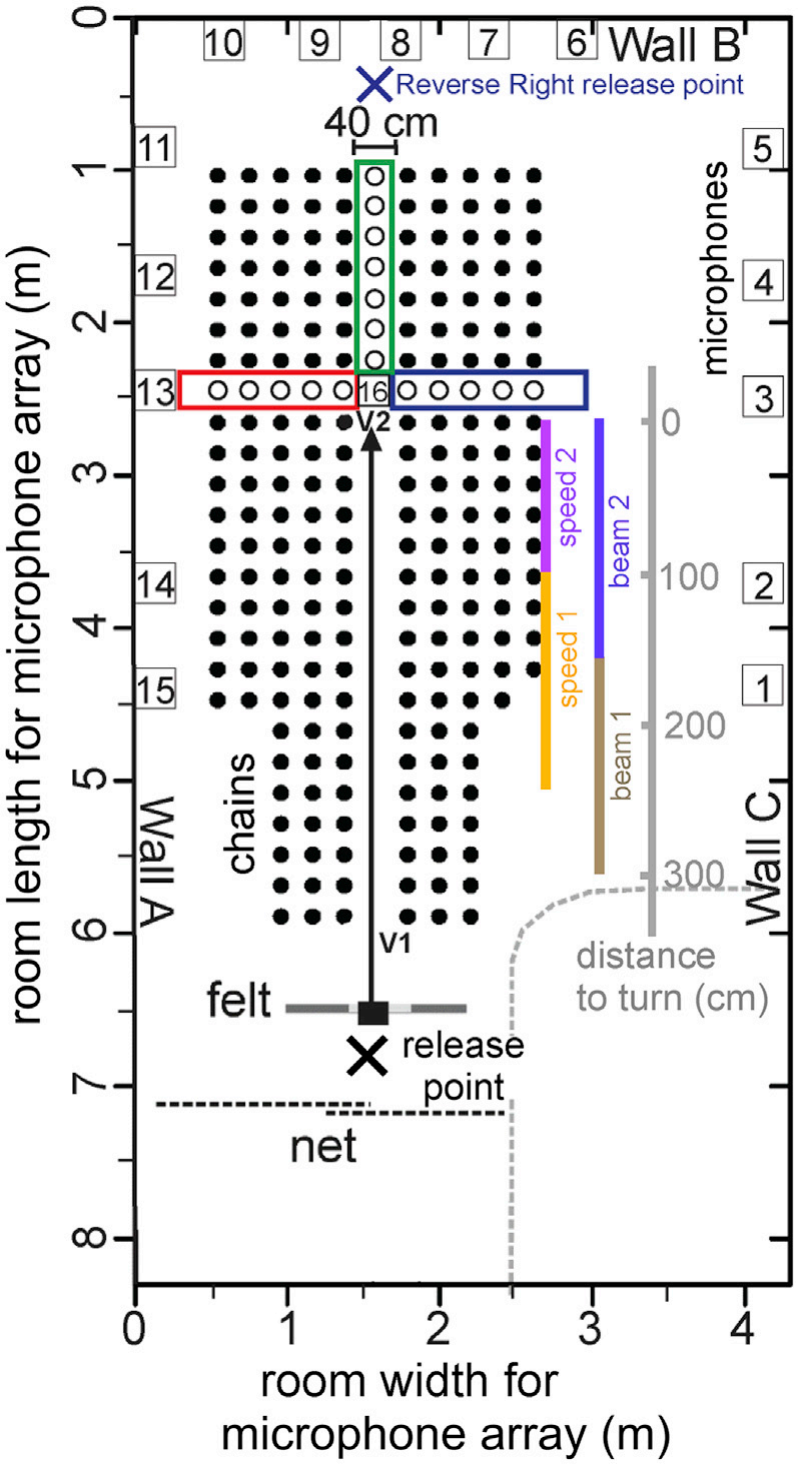
Diagram of the flight room and chain array Dimensions of the flight room (m; left y and x axes) and the location of the hanging chains (filled circles) are shown. Bats were released through an opening in hanging felt (small filled rectangle) at the release point (black X) and flew down a corridor (40 cm width) through the chain array. Chains were hung in rows and columns 20 cm apart. In the Straight condition, chains were removed (open circles) from the section outlined in green, and both the red and blue sections were blocked with chains. Bats were rewarded for landing on Wall B. In the Right Turn condition, chains were removed from the blue section of the chain array, and the green and red sections were blocked with chains. Bats were rewarded for landing on Wall C. In the Left Turn condition, chains were removed from the red section, and the blue and green sections were blocked with chains. Bats were rewarded for landing on Wall A. In the Reversed Right Turn condition, bats were released from the blue X on Wall B and flew through the green and red sections, landing on Wall A. The blue section was blocked by chains. The colored lines on the right side of the array marked beam 1 (brown) and beam 2 (dark purple) show the two segments of the flightpath used for calculating beam aim. These two segments are referred to distance from the turn, as shown by the gray numbers; the turn is at 0 cm and the beginning of measured flightpath is at 300 cm. The colored lines marked speed 1 (orange) and speed 2 (purple) show the two segments of the flightpath used to calculate flight speed. Small numbered boxes around the perimeter of the room show the locations of recording microphones, and boxes labeled “v” show the position of recording cameras. Black dotted lines indicate a net that prevented the bat from flying into the rest of the room, and recording equipment was located behind the curved gray dotted line.

Four big brown bats were challenged to fly through a 40-cm-wide corridor surrounded by an array of 217 hanging plastic chains that produced intense, extended echoes (acoustic clutter; see transparent methods). Two of these corridors incorporated sharp 90° right or left turns into the flightpath (Figure 1). The task required the bat to detect the open gap in the chain array and then perform an abrupt flight maneuver to navigate through it and receive a reward. Bats completed 7 days of flights in the Straight corridor condition, 9 days in the Right Turn condition, and 6–7 days in the Left Turn condition. Two bats (Bat 3 and Bat 4) also completed 1 day of flights in a Reversed Right Turn condition.

### Performance

**Figure 2:**
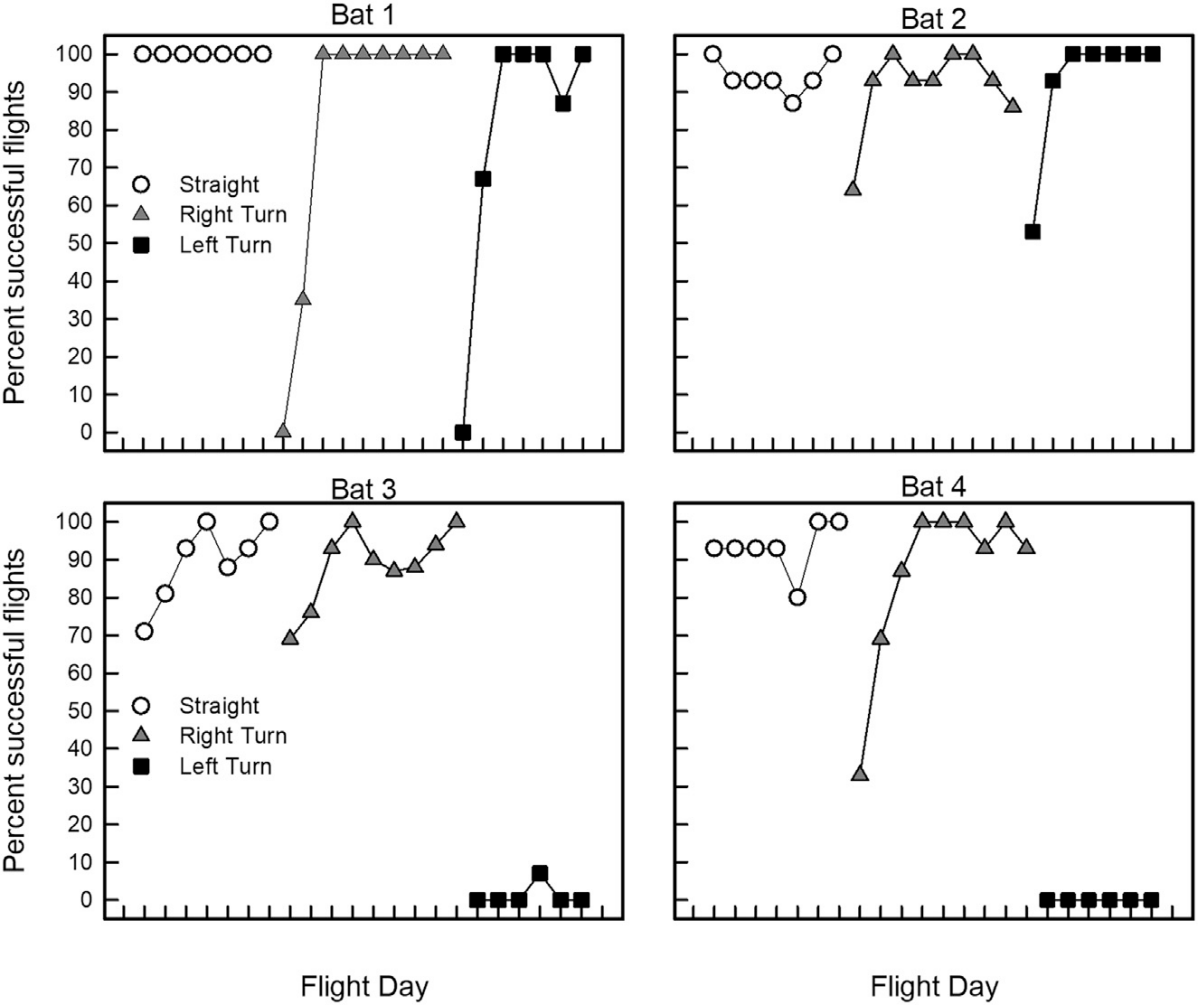
Performance of individual bats on each flight day in the three different task conditions Bats performed 15–18 flights per day. All bats showed improved performance (increased percentage of successful flights) with experience in Right Turn flights; only two bats showed improved performance with experience in Left Turn flights.

We defined a successful flight as one in which the bat flew the entire corridor length to the appropriate wall without turning around or exiting the corridor through the chains, landing on a chain, or falling to the floor. Percent successful flights (Figure 2) varied significantly with task condition [one-way analysis of variance: F(2,42) = 34.21, p < 0.001] and among bats [F(3,21) = 13.09, p < 0.001]. All bats flew through the Straight corridor with few errors [mean (M) percent correct = 94.2%, N = 420 flights]. Over seven days of flights, the mean success rate increased from 91% on the first day of flights to 100% on the last day of flights. Performance of previously experienced (Bats 1 and 4) and previously naive (Bats 2 and 3) was similar (M = 96.7% and 91.8%, respectively).

Performance in the Right Turn condition (M = 86.9%, N = 545 flights) did not differ significantly from that in the Straight condition (pairwise comparisons: p = 0.096). All four bats, regardless of whether they had previous laboratory experience in flying amid clutter, experienced a large drop in performance on the first day of the Right Turn condition, with success rate ranging from 0% to 69% (M = 45.1%). Success rate reached M = 84.8% within the first 3–4 days of this condition (Figure 2). Experienced and naive bats had similar success rates (M = 83.9% and 89.9%, respectively).

Success rate in the Left Turn condition differed considerably among bats. Bat 1 and Bat 2 improved their performance over flight days, with success rate increasing from M = 26.5% on the first day of flights to M = 100% on the last day of flights. On the other hand, neither Bat 3 nor Bat 4 completed the task successfully, with success rates of 0% on both the first and the last day of flights.

We statistically analyzed performance on a bat-by-bat basis. Bat 1 and Bat 2 both showed similar performance across conditions [F(2,18) = 1.13, p = 0.35; F(2,20) = 0.11, p *=* 0.90, respectively]. Conversely, Bat 3's performance differed significantly across conditions [F(2,19) = 204.89, p < 0.001]. This bat performed equally well in the Straight and Right Turn conditions (pairwise comparisons: p = 0.85), but significantly worse in the Left Turn compared with Right Turn condition (pairwise comparisons: p < 0.001). Bat 4's performance also differed significantly across conditions [F(2,19) = 78.06, p < 0.001]. This bat performed similarly in the Straight and Right Turn conditions (pairwise comparisons: p = 0.36), but significantly worse in the Left Turn compared with the Right Turn condition (pairwise comparisons: p < 0.001).

Bat 3 and Bat 4 completed 1 day (N = 39 flights) in the Reversed Right Turn condition, with a success rate of 80% correct. Because of the small sample size in this condition, these data were not included in subsequent statistical analyses.

### Beam aim

**Figure 3:**
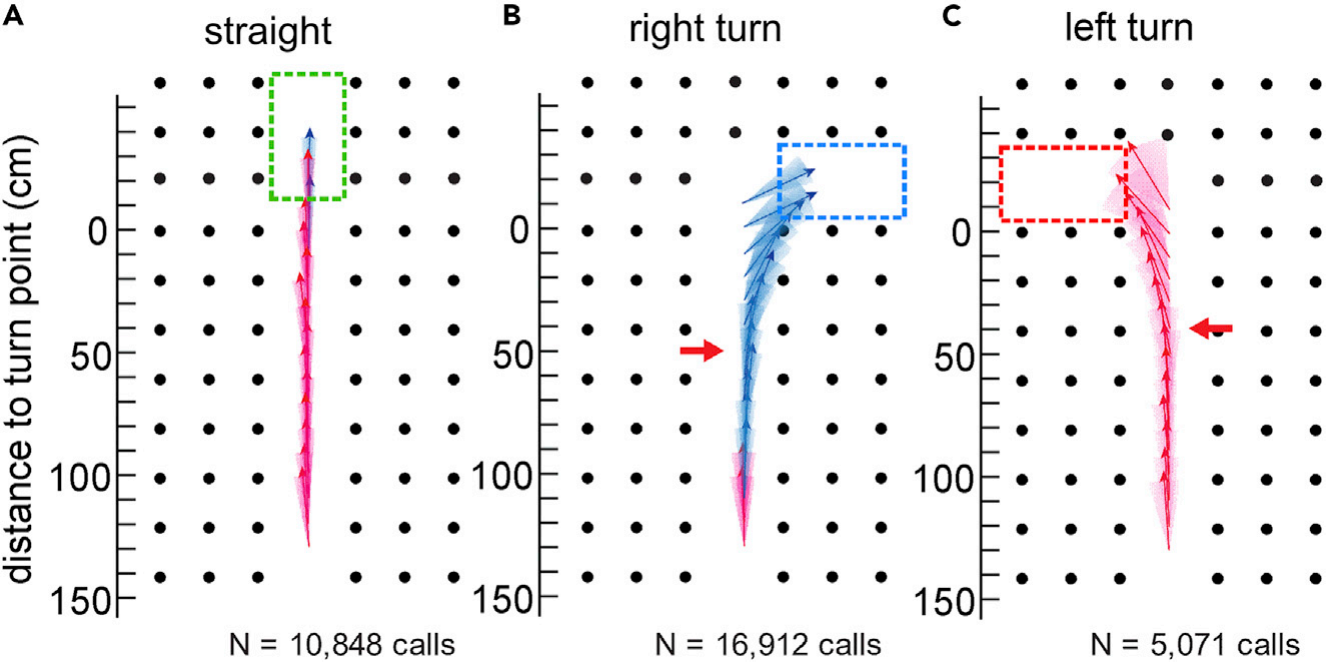
Mean and standard deviation (SD) of beam aim of all calls emitted in successful flights (A–C) (A) Straight flights; (B) Right Turn flights; (C) Left Turn flights. The y axis shows the portion of the flightpath (see beam 2 line, Figure 1) immediately before the turn (or equivalent point in Straight flights). The position of the turn (or equivalent location in straight flights) is shown by the green, blue, and red dashed rectangles in (A, B, and C), respectively. Filled circles show the positions of hanging chains in each condition. Colored vectors plotted along the midline of the chain array show mean beam aim calculated in 10-cm bins. Colored envelopes around these vectors show the SD within each window. Blue vectors denote positive beam aims >0°, red vectors denote negative beam aims <0°. N = Numbers of calls used to calculate beam aim. In (B and C), red horizontal arrows denote the area in which beam aim diverges more than 1 SD from the midline.

We quantified beam aim using cross-correlation and time-difference-of-arrival of calls recorded by 14 microphones (see transparent methods). We calculated beam aim angles (M, SD; Table 1) in successful flights by individual bats in each flight condition for two portions of the flightpath (Figure 1): 300 to 150 cm before the turn (beam 1, brown) and 150 to 0 cm before the turn (beam 2, dark purple). Mean (M) beam aim was calculated in 10-cm bins. Mean beam aims (+/− 1 SD) in all successful Straight, Right Turn, and Left Turn flights are plotted in Figures 3A–3C.

**Table 1:** Beam aim angles in all successful flights for each bat

Condition	Distance to turn		Bat 1	Bat 2	Bat 3	Bat 4
**Straight**	300 cm–150 cm	**N**	*1,895*	841	1,101	*1,744*
		**M**	*−1.25°*	0.39°	1.74°	*3.27°*
		**SD**	*8.12°*	6.73°	5.98°	*8.23°*
	150 cm–0 cm	**N**	*1,222*	696	775	*975*
		**M**	*−3.87°*	−3.66°	−0.43°	*0.77°*
		**SD**	*6.17°*	6.40°	6.13°	*9.50°*
**Right Turn**	300 cm–150 cm	**N**	*2,464*	648	1,522	*2,735*
		**M**	*3.95°*	−2.62°	2.37°	*2.38°*
		**SD**	*8.85°*	6.91°	6.69°	*7.39°*
	150 cm–0 cm	**N**	*2,082*	937	1,929	*1,953*
		**M**	*27.88°*	16.49°	28.11°	*25.08°*
		**SD**	*27.55°*	27.61°	27.08°	*27.07°*
**Left Turn**	300 cm–150 cm	**N**	*1,695*	379	–	
		**M**	*−3.46°*	0.17°	–	–
		**SD**	*12.52°*	9.78°	–	–
	150 cm–0 cm	**N**	*1,506*	656	–	
		**M**	*−26.55°*	−20.56°	–	–
		**SD**	*26.74°*	18.77°	–	–

N = number of angles, M = mean, SD = standard deviation. Flightpath is divided into two: 300–150 cm (beam 1, brown line, Figure 1) and 150–0 cm (beam 2, dark purple line, Figure 1) before the 90° turn (or equivalent for Straight flights). Empty cells indicate no data for that combination of bat and condition. Italicized values are from the two bats that were experienced in flight tasks.

In the Straight condition (Figure 3A), beam aims of all bats remained centered around 0° throughout the flightpath, with a mean deviation from midline toward the left of −1.03° (beam 1) and −1.79° (beam 2). There is no statistical difference in beam angle between the two experienced and the two naive bats for either beam 1 or beam 2 (Welch two-sample t test, p = 0.14 and 0.62, respectively). In the Right Turn condition (Figure 3B, Table 1), mean angles for beam 1 were +1.52° away from midline toward the right; this increased to a mean of +24.39° for beam 2, as the bats aimed their beams toward the right edge of the corridor. The two experienced bats had larger beam angles (i.e., shifted more toward the right) than the two naive bats (p < 0.0001 for both beam 1 and beam 2). We then examined when during the 150–0 cm segment of the flightpath (beam 2) that bats first began to aim toward the Right Turn. We chose as the criterion the first point at which the beam angle at beam 2 was more than 1 SD away from the mean angle for beam 1. These rightward shifts occurred at 60 cm (Bat 1), 30 cm (Bat 2), 60 cm (Bat 3), and 65 cm (Bat 4) from the turn point. In the Left Turn condition (Figure 3C, Table 1), beam aim 1, averaged over the two successful bats, was −1.65° toward the left edge of the corridor. Mean angle for beam 2 increased to −23.55° toward the left. The experienced bat (Bat 1) had larger beam angles (i.e., more to the left) than the naive bat (Bat 2) for both beam 1 and beam 2 (p < 0.001). Using the same criterion described above, beam aim shifted toward the left 40 cm (Bat 1) and 60 cm (Bat 2) before the bat physically reached the turn.

Beam aim angles on failed flights are summarized in Table S1. In Straight failed flights, beam aim angles over all four bats are larger (M = 5.29° and 3.45°, beam 1 and beam 2, respectively), indicating greater deviation from the midline, than those in successful flights (Table 1). Beam angles in failed Right Turn flights for beam 1 was close to the midline (M = +1.45°) and increased to M = +21.86° for beam 2. This shift to the right is smaller than that in successful Right Turn flights (Table 1), indicating that bats did not aim their beams as effectively toward the right edge of the corridor. In failed Left Turn flights, beam aim averaged over all four bats remained at the midline (M = +0.49°) for beam 1, but switched to the right, rather than the left, side of the corridor for beam 2 (M = +15.63°). In other respects, beam aim was part of the overall behavior of failed flights, which entailed the bat not flying along the corridor but leaving it or landing on the floor, which coupled flight and beam in a manner difficult to untangle.

**Figure 4:**
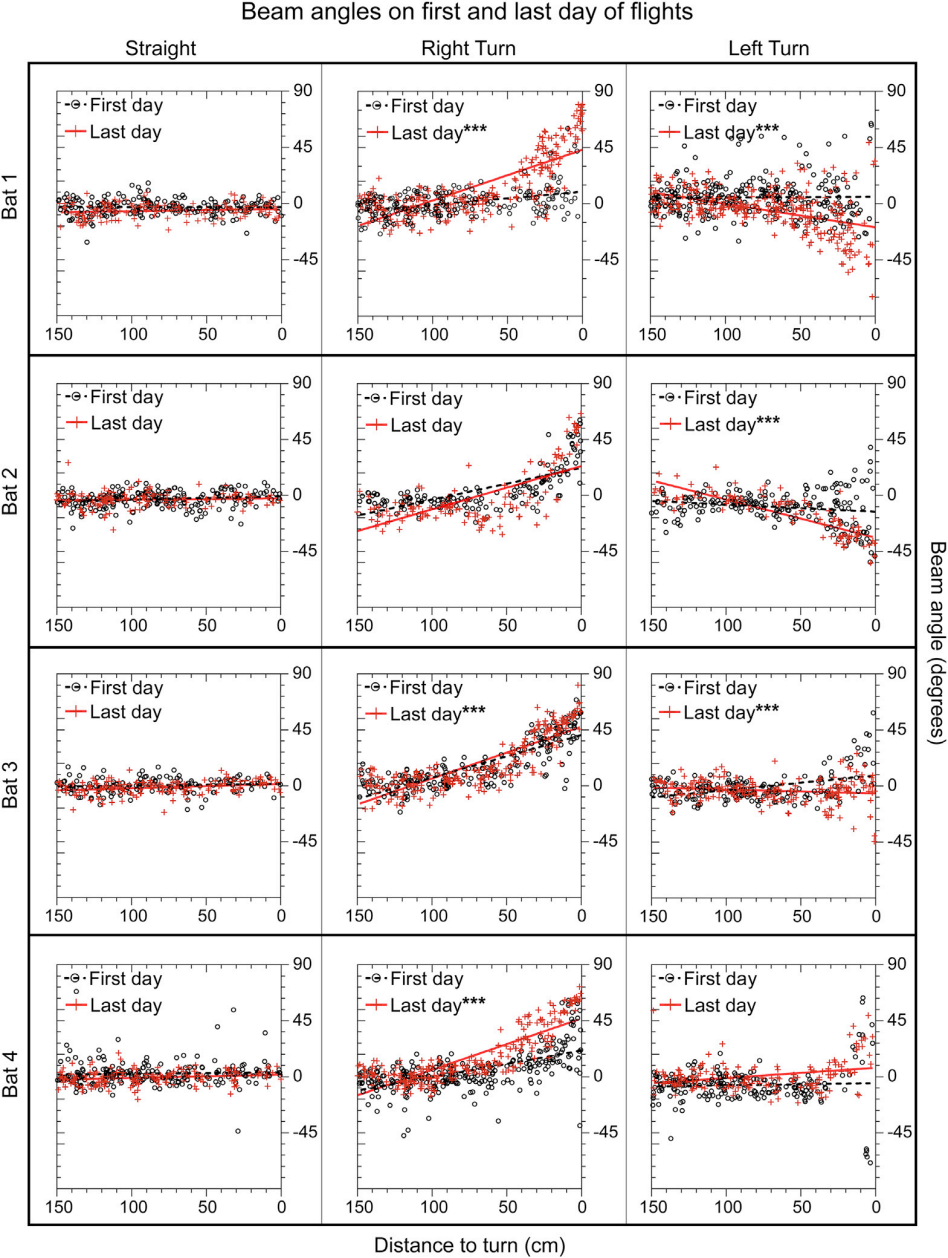
Beam angle of calls emitted from 150–0 cm before the turn (or equivalent location in Straight flights) on first and last day of each flight condition Each panel shows beam angles for the first day of flights in that condition (open black circles), beam angles for the last day of flights in that condition (red crosses), and regression lines (first day, black; last day, red) through the data points. Distance to the turn (150–0 cm) is shown on the x axis. y Axis shows beam angle (positive values show shifts toward the right, negative values show shifts toward the left). Asterisks next to “Last day” indicate that beam aim regression in the final day was significantly different from that in the first day. Plots show beam aim values from both successful and failed flights, with the exception of Left Turn flights for Bats 3 and 4, which show values from failed flights only.

We then asked if beam aim before the turn (150–0 cm, beam 2) differed between the beginning and the end of flights in the three conditions. We plotted the beam aim of all calls in this portion of the flightpath for all flights (both successful and failed) on the first day and on the last day of each condition (Figure 4). Beam angles in failed flights are plotted in Figure S1. We calculated the linear regression through the data from all flights on each of these 2 days and compared the slopes of the regression lines. In the Straight condition, beam aim slope did not differ significantly (Bonferroni-corrected α = 0.004) between the first and last day of flights for any bat [Bat 1: F(1,330) = 1.6, p = 0.21; Bat 2: F(1,325) = 0.94, p = 0.33; Bat 3: F(1,267) = 1.30, p = 0.26; Bat 4: F(1,352) = 0.54, p = 0.46]. Bats aimed their beam within ±5° of the midline on both days.

All bats successfully navigated the Right Turn. On the first day of Right Turn flights, Bat 1 emitted most calls straight ahead, centered around 0° (Figure 4, center column). On the final day, Bat 1 shifted its beam aim toward the right, with the regression intercept at +45° [significantly different than the first day: F(1,467) = 107.8, p < 0.0001]. Bat 2's beam aim did not differ significantly between the first and last days of Right Turn flights [F(1,272) = 5.2, p = 0.02], because its beam on the first day of flights had already shifted toward the Right Turn. Bat 3 also shifted its beam toward the right on the first day of Right Turn flights, and showed even more pronounced shifts on the last day, to an angle of +45° [F(1,408) = 8.7, p = 0.003]. Bat 4 aimed its calls on the first day toward the right (regression intercept of +21°), with a more pronounced shift on the last day [regression intercept of +45°; F(1,497) = 61.7, p < 0.0001]. Note that on the final day of Right Turn flights, three bats continued to emit a small number of calls toward the front (±10°) or to the left (<−10°) within 30 cm of the Right Turn (Bat 1: 11 calls, Bat 2: 6 calls, Bat 3: 1 call, Bat 4: 0 calls). Even on failed Right Turn flights (Figure S2), the beam is generally aimed toward the right, with considerable scatter.

Differences in performance among bats in the Left Turn condition are reflected in their beam aims (Figures 4 and S1). The third column in Figure 4 shows the calls emitted by each bat on the first day after switching from the Right Turn to the Left Turn condition. Bat 1 emitted its calls toward the center of the corridor on the first day of flights, whereas on the final day its beam aim had shifted in the direction of the Left Turn, at an angle of −19° [significantly different from the first day: F(1,555) = 39.3, p < 0.0001]. Bat 2 also emitted its calls toward the center during the first day of Left Turn flights, whereas on the final day it shifted its beam toward the left at an angle of −36° [F(1,285) = 52.6, p < 0.0001]. In contrast, Bat 3 emitted most of its calls toward the right on the first day, at an angle of +9°, and shifted to the left at an angle of −6° on the final day [F(1,376) = 36.6, p < 0.0001]. This shift was toward the correct direction, but it was not large enough to guide the bat through the Left Turn successfully. Bat 4 aimed its beam slightly toward the left (−9°) on the first day of this condition, but on the last day it aimed its beam to the right at an angle of +9° [not significantly different: F(1,347) = 3.2, p = 0.07]. This failure to switch beam aim to the appropriate direction (see also Figure S1) is consistent with this bat's behavioral failure to navigate the Left Turn (Figure 2). All bats directed some calls straight ahead (±10°) or to the right (>10°) within 30 cm of the turn on the last day of the Left Turn condition (Bat 1: 20 calls, Bat 2: 1 call, Bat 3: 43 calls, Bat 4: 25 calls).

### Flight speed

We measured bats' flight speeds (see transparent methods) over the portion of the flightpath before they reached the turn (or equivalent point in Straight flights). We separated this portion into two—a farther segment (250–100 cm before the turn; speed 1, orange line, Figure 1) and a nearer segment (100–0 cm before the turn; speed 2, purple line, Figure 1). The nearer segment (0–100 cm before the turn) was chosen for flight speed measurements because individual bats diverted their beam angle away from the midline at various points between 0 and 100 cm from the turn (see Figure 4), indicating that the bats began physically preparing for the turn within 100 cm from the turn. The farther segment (250–100 cm) was chosen as the comparison range, as this segment was most representative of the bat's stable flight speed in the straight section of the turn conditions; distances farther than 250 cm from the turn were excluded to avoid variable flight speeds due to the bat's initial release. Median flight speed in the farther segment of the Straight corridor differed among bats, from a high of 3.1 m/s for Bat 1 to a low of 2.6 m/s for Bat 3. All bats flew slower in the farther segment of the Turn corridors, with median flight speeds of 2.4 to 2.2 m/s (Figure S2). Flight speeds in the nearer segment of the Straight corridor were consistently lower than in the farther segment, varying from a high of 2.9 m/s for Bat 1 to a low of 2.2 m/s for Bat 3. All bats significantly decreased flight speeds (to 1.8–2.0 m/s) in the nearer segment of the Turn corridors (Kolmogorov-Smirnov tests, p < 0.001). Flight speeds could not be calculated in failed flights, because bats aborted these flights at different locations in the corridor.

### Call timing

We calculated inter-pulse intervals (IPI), the time between each individual pulse, for all calls emitted during flights. To quantify how the bats modified their call timing in the three flight conditions, we fit IPI values from successful flights to two separate linear mixed effects models (LMMs). We also categorized calls into SSGs to compare pulse clustering in the three conditions. We plotted IPI data from both successful and failed flights as distributions of pre- and post-IPI in order to highlight qualitative differences in the timing strategies used by individual bats in relation to their behavioral performance.

### Linear mixed effects models

**Figure 5:**
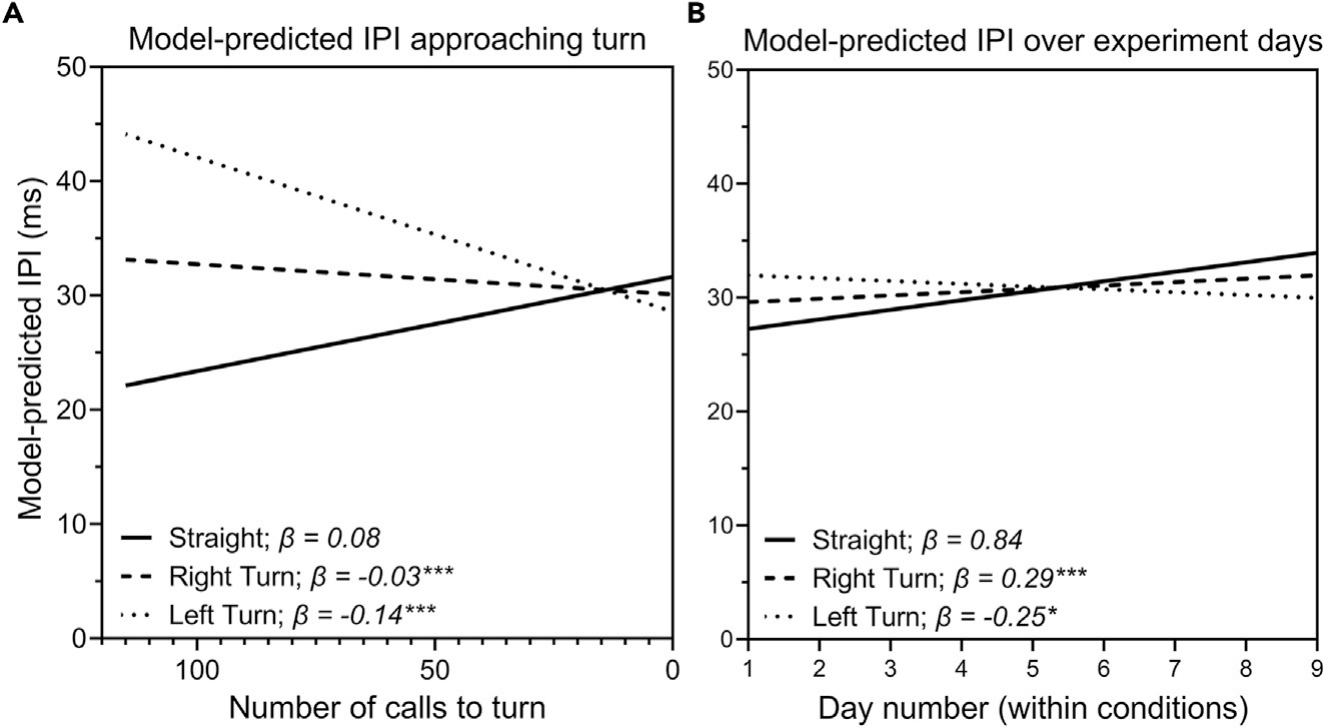
Results of linear mixed effects models (A and B) Linear mixed effects model predictions of changes in IPI in successful flights as the bat approaches the turn (A) and as the bat completes multiple days of flights in the same condition (B). Asterisks indicate that slopes for the turn flights are significantly different from the slope for Straight flights.

We predicted that bats would decrease IPIs as they approached the turn point, as the area, particularly the depth, that is acoustically visible to them shrinks. We also predicted that IPIs would increase over time within each of the three flight conditions, as the bats became more accustomed to the spatial layout and less dependent on perceptual information regarding their immediate surroundings. To test these predictions, we fit the IPI timing data (defined as the post-IPI value, i.e., the time interval after each pulse) to two LMMs. The first LMM tested whether linear relationships exist in how IPIs changed as the bats approached the turn (or the equivalent point in Straight flights), as well as how these relationships differ among individual bats and different task conditions. Only IPIs of calls emitted before entering the turn (or the equivalent location in Straight flights) were input into the model. Three fixed effects were included: Condition (Straight, Right Turn, or Left Turn), Number of Calls, and the interaction of those two effects. The variables Trial Number, Day number, Condition, and Bat were added as nested random effects. Table 2 shows the results of this model, and Figure 5A shows the model's predicted regression lines of change in IPI as the bats approached the turn (or equivalent point) in each condition. IPI values in successful flights did not differ significantly among conditions (p > 0.05). Mean IPIs from each individual bat varied from 27–35 ms in the Straight condition, 27–28 ms in the Right Turn condition, and 25–41 ms in the Left Turn condition. (In contrast, over all failed flights by all bats, mean IPI ranged between 25 and 27 ms). The results of the LMM showed a significant interaction between Condition and Number of calls (p < 0.001; Table 2). IPI increased in the Straight condition as the bat approached the equivalent point where the turn was located in future conditions, by 0.08 ms per call (Figure 5A). Conversely, in the Right Turn condition, IPI decreased by 0.03 ms per call (significantly different than the increase in the Straight condition, p < 0.001). In the Left Turn condition, IPI decreased even more, by 0.14 ms per call (significantly lower than in both the Straight and Right Turn conditions, p < 0.001).

**Table 2:** Results of the linear mixed effects model of IPI as the bat approaches a turn

Linear mixed effects model predicting post-IPI as bat approaches turn			
Predictors	Estimates	CI	p
(Intercept)	33.17	26.94–39.40	**<0.001**
Condition	−1.53	−3.82–0.75	0.189
Number of calls to turn	−0.19	−0.21–−0.17	**<0.001**
Condition × No. of calls	0.11	0.10–0.12	**<0.001**
Random effects	Variance		
σ^2^	141.56		
Trial number	4.72		
Day number	3.14		
Condition	6.28		
Bat	20.74		
Total Observations	176.44 46,901		

CI, confidence interval. P values in bold are statistically significant.

To determine whether the non-randomized order of flight conditions affected the results, we fit the IPI values to a second LMM, which analyzed the change in IPI as a factor of the number of experimental days the bat spent in that condition. This LMM includes fixed effects for Number of days and for the interaction between Condition and Number of days. Table 3 shows the results from this LMM, and Figure 5B shows the model-predicted IPI over the nine flight days within the three consecutive conditions. There is a significant effect of condition (p < 0.05) and a significant Condition by Number of days interaction (p < 0.001; Table 2). IPIs in Straight flights increased significantly over days, by 0.84 ms per day (p < 0.001). In the Right Turn condition, IPIs also increased over days, by 0.29 ms, but significantly less than the increase in Straight flights (p < 0.001). Conversely, IPIs decreased by 0.25 ms per day in Left Turn flights (significantly different from Straight flights, p < 0.05).

**Table 3:** Results of the linear mixed effects model of IPI over consecutive days within conditions

Linear mixed-effects model predicting post-IPI over experimental days			
Predictors	Estimates	CI	p
(Intercept)	23.51	17.54–29.48	**<0.001**
Condition	2.89	0.76–5.02	**<0.05**
Day number	0.84	0.88–1.88	**<0.001**
Condition ∗ Day No.	−0.54	−0.80–−0.28	**<0.001**
Random effects			
σ^2^	142.86		
Trial number	4.39		
Day number	1.72		
Condition	4.08		
Bat	20.12		
Observations	46,901		

CI = confidence interval. P values in bold are statistically significant.

**Figure 6:**
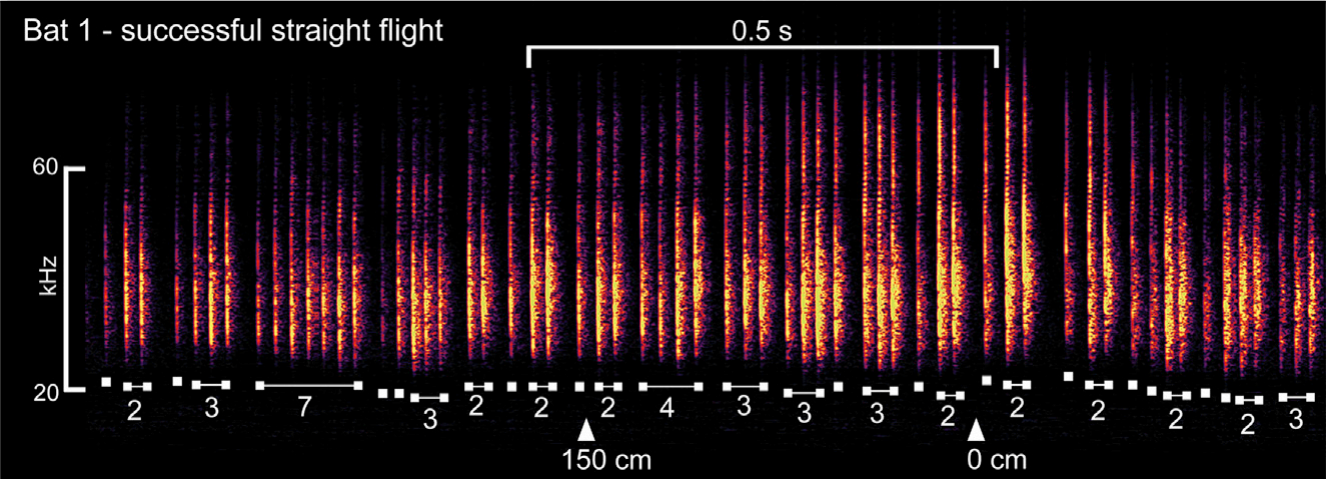
Spectrogram of calls emitted by Bat 1 during a single successful flight through the Straight corridor SSGs were categorized using the algorithm devised by Kothari et al. (2014) and modified by Warnecke et al. (2016, 2018). White labels and numbers underneath calls identify these SSG categorizations and the number of pulses within each group. White triangles show the bat's position relative to the point at which the position of the turn (at 0 cm) in the Turn conditions (beam 2 in Figure 1). After flying past the 0 cm mark, the bat lowers the frequency of its pulses in preparation for landing on the wall.

Although the LMMs allow us to determine if there are linear trends over time or among conditions, they do not give detailed information about more complex, nonlinear temporal calling patterns. This includes the grouping of calls into SSGs, groups of two or more closely spaced calls flanked by longer IPIs. To categorize the bat's clustering of calls into groups, we implemented a previously described algorithm (Kothari et al., 2014) with revised criteria as used in experiments that included high-density clutter (Warnecke et al., 2016, 2018). An example of the output of this algorithm is shown in Figure 6 (successful Straight flight) and Figure S3 (successful and failed Right Turn flights).

**Figure 7:**
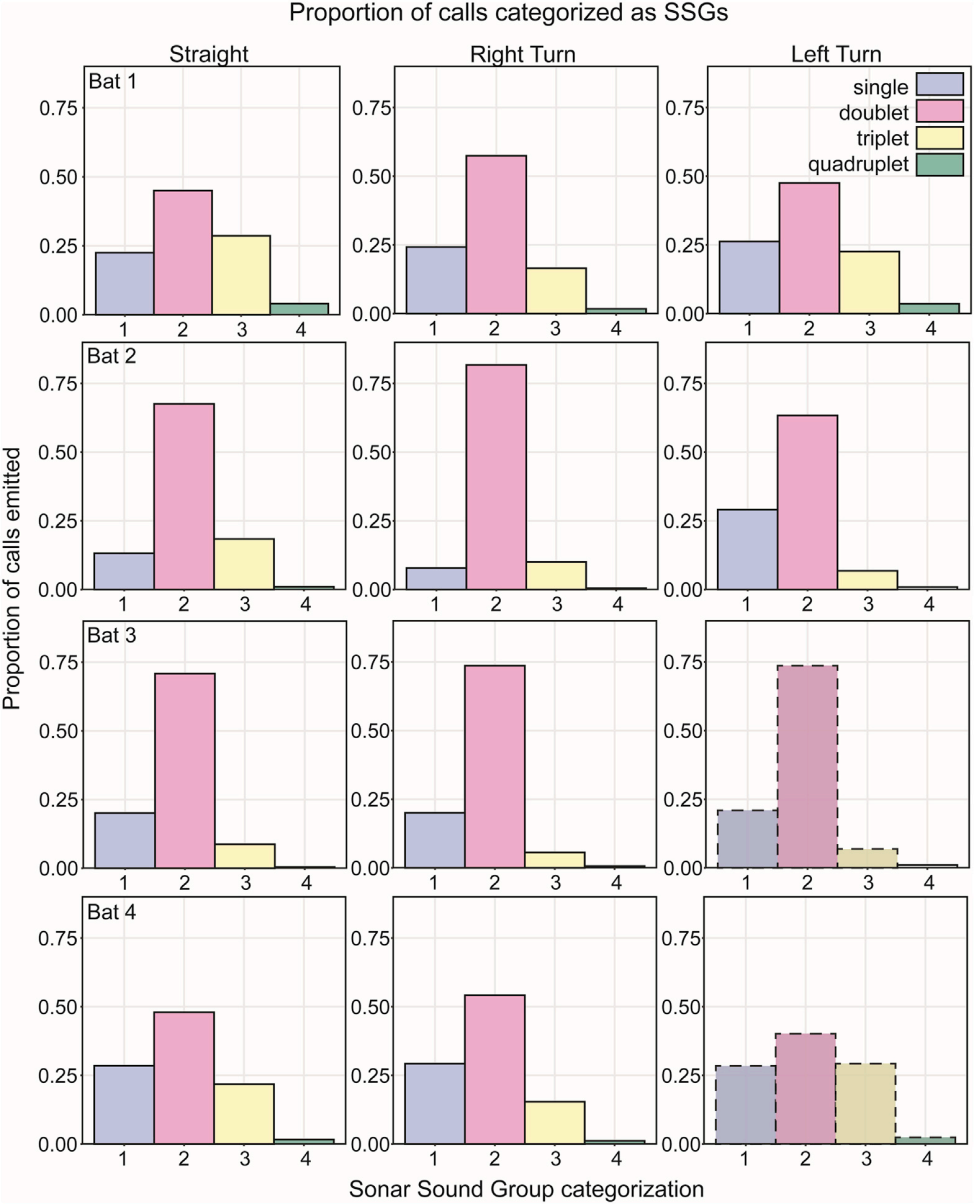
Proportions of calls emitted as Sonar Sound Groups (SSGs) Bats emitted the majority of their calls in SSGs (as opposed to single calls) in all flight conditions, although the relative proportion varies. SSG of 1 (blue) = single call; 2 (pink) = doublet; 3 (yellow) = triplet; 4 (green) = quadruplet. Solid outlined bars show data from successful flights. The dashed outlined bars (Bat 3 and Bat 4, Left Turn) show failed flights. SSG/single call proportions in Turn flights are significantly different from SSG/single call proportions in Straight flights for all bats (McNemar chi-square tests, p < 0.001).

We calculated the proportions of SSGs in successful and failed flights in all three flight conditions. Figure 7 and Table S2 show the relative proportion of calls in each condition identified as single calls or as SSGs of varying size. Data are shown for successful flights only, except for Bat 3 and Bat 4 in the Left Turn condition, which show data from failed flights. Bats emitted most of their calls in SSGs containing two or more calls as they approached the turn (or equivalent point in the Straight condition). The most common SSG was a doublet (proportions of 0.393–0.817), with triplets or single calls the second most common. A few calls were classified as SSGs of 7 or 8 pulses. In successful flights, changes in condition from Straight to Turn led to significant increases in the proportions of calls emitted as SSGs [Bat 1: Straight versus Right, χ^2^_(1, N = 9732)_ = 656, p < 0.001; Bat 1: Straight versus Left, χ^2^_(1, N = 8683)_ = 815, p < 0.001; Bat 2: Straight versus Right, χ^2^_(1, N = 8458)_ = 1,906, p < 0.001; Bat 2: Straight versus Left, χ^2^_(1, N = 5267)_ = 1,605, p < 0.001; Bat 3: Straight versus Right, χ^2^_(1, N = 10791)_ = 589, p < 0.001; Bat 4: Straight versus Right, χ^2^_(1, N = 11317)_ = 407, p < 0.001]. Consistent with the data from successful flights, the majority of SSGs in failed flights (Table S2; Bat 3 and Bat 4, Left Turn) were doublets. Bat 3 and Bat 4 emitted small proportion of pulses (0.004–0.041) in groups of 4 and 5, and Bat 4 emitted some groups of 6 and 7. For these two bats, the proportion of SSGs differed significantly between Right Turn and Left Turn conditions [Bat 3: Right versus Left, χ^2^_(1, N = 12586)_ = 2,825, p < 0.001; Bat 4: Right versus Left, χ^2^_(1, N = 13244)_ = 1,247, p < 0.001], with the difference due to production of more SSGs of 3–8 pulses.

**Figure 8:**
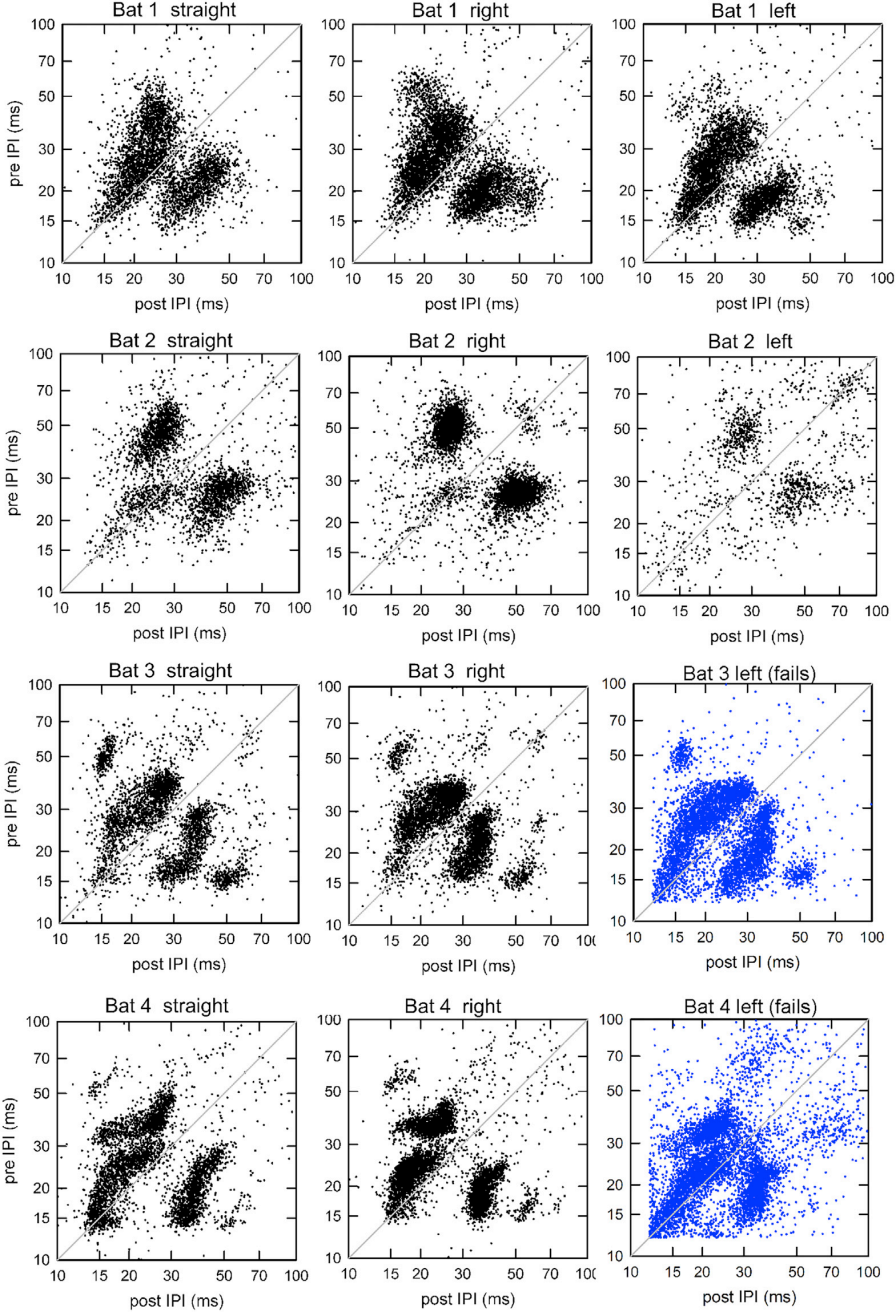
The distribution of pre-IPI and post-IPI of all calls emitted in all flights Calls along the gray solid diagonal line have equal pre- and post-IPI values. Plots with blue symbols show IPIs in failed Left Turn flights for Bat 3 and Bat 4. Bat 2 in the Left Turn condition emitted fewer calls, so the plot seems less dense. These plots reveal identifiable and consistent qualitative differences in the timing patterns that individual bats employ when navigating through clutter. Note persistence of distinctive plot patterns for each bat across conditions.

The non-linear way in which bats modify the timing of their calls is also visualized by plots of the distribution of pre-IPI values of every call against the corresponding post-IPI values (Figure 8). These plots serve as a form of visual “fingerprint” highlighting the unique temporal pattern of call timing that each individual bat employed, despite all bats navigating the same physical environment. They are useful for displaying pulse clustering because they do not rely on a specific algorithm for defining SSGs; as shown previously, changing the algorithm produces different classifications (Kothari et al., 2014; Warnecke et al., 2016, 2018). Figure 8 shows that individual bats emitted calls with both long (>50 ms) and short (<50 ms) IPIs, but varying in such a way as to form dark clusters of values above and below the diagonal line (where pre-IPI = post-IPI). The diagonal line shows a 1:1 relationship between pre-IPIs and post-IPIs. Clustering of IPIs away from the diagonal shows that SSGs are present, although these plots cannot identify specific types of SSGs. Comparing plots between individual bats flying in the Straight condition (Figure 8, first column) shows that these clusters differ in their number and position across bats. Note that for Bat 3 and Bat 4, the clustering of pulses in their failed flights is similar to that in their successful flights (Figure 8, last column). These temporal fingerprints are consistent between flight conditions for an individual bat, although they do differ among bats.

## Discussion

### Turning amid clutter is a difficult task

This experiment required big brown bats to navigate down narrow corridors and through sharp 90° turns embedded in intense acoustic clutter produced by a dense array of surrounding chains. We hypothesized that bats would find this task challenging, and our results support this hypothesis. Bats were able to fly down Straight corridors with a mean success rate of 94% (four bats). This high success rate is consistent with those found in other experiments that also challenged bats to navigate narrow straight or curved corridors surrounded by various densities of clutter (mean success rates of 93% [Hom et al., 2016], 94% [Simmons et al., 2018], 97% [Wheeler et al., 2016], and 95%–99% [Warnecke et al., 2016, 2018]). Adding abrupt 90° turns to the flightpaths decreased mean success rates to 84% (four bats) in the Right Turn condition and to 85% (two bats) in the Left Turn condition. A previous experiment challenging bats to fly through 40-cm openings in fine mist nests reported success rates of 50%–82% (Surlykke et al., 2009). In that experiment, bats were not required to make sharp turns, and the mist nets returned echoes that were less intense than those reflected by the chains in our experiment. Another experiment in which bats had to navigate past fine mist nests reported excellent performance (Knowles et al., 2015).

Bats were initially unable to adapt to a sudden change in flightpath, from Straight to Right Turn or from Right Turn to Left Turn, requiring 2 to 3 days (30–45 individual flights) to achieve final success. In the Right Turn condition, performance improved for all bats, reaching 100% correct in one animal, as they gained more experience with the flightpath over experimental days. After initial failures, two bats were able to achieve a success rate of 100% in the Left Turn condition; however, two other bats were unable to master that condition over the duration of the experiment, even with practice. These large individual differences indicate that the Left Turn was not intrinsically more difficult due to its closer proximity to the flight room wall. Moreover, bats' previous experience with flying down narrow corridors does not explain this individual variability; the same bat that achieved a high level of performance in earlier corridor experiments (Wheeler et al., 2016) had a success rate of 0% in the Left Turn condition, whereas a previously naive bat achieved a final success rate of 85%. It is possible that the two bats that failed to navigate the Left Turn could have learned it with even more experience, but this could not be tested as they were unwilling to continue flying in that condition. These same two bats were able to navigate a Reversed Right Turn condition, which attempted to control for proximity of the turn to the wall. However, this condition was run for only one day, rather than the 7–9 days of the other conditions, again due to the bats' refusal to continue flying. We cannot determine if difficulty in the Left Turn condition might relate to the bats' handedness (Zucca et al., 2010).

### Beam aim anticipates the turn

A previous experiment quantifying beam aim required big brown bats to fly through an open room or through openings in mist nets to catch a tethered insect prey (Ghose and Moss, 2003; Surlykke et al., 2009). In the open room, bats focused their beam aim on the prey about 300 ms before reaching it, with a beam aim accuracy of around 3° (Ghose and Moss, 2003). When mist nets were introduced, bats sequentially scanned the edges of the openings, with an accuracy of ±5°, about 100–300 ms before flying through the opening (Surlykke et al., 2009). Bats flying through the Straight corridor in our experiment aimed their sonar beam straight ahead relative to their heading at a similar accuracy.

We hypothesized that bats flying toward abrupt 90° turns would aim their sonar beam in the direction of these turns before reaching them. Our results support this hypothesis. Bats that were able to navigate in the Turn conditions shifted their beam in the appropriate direction between 30 and 65 cm (224–359 ms) before reaching the turns. These anticipatory times are at the upper edge of the time range quantified by Surlykke et al. (2009). We suggest that these longer anticipatory times reflect the increased difficulty of locating the gap in the dense, intense acoustic clutter surrounding it. Even in these successful flights, bats continued to aim a small number of calls straight ahead or toward the opposite side of the corridor, but there was little sequential side-to-side scanning. This pattern, measured from 32,831 biosonar calls, was remarkably consistent across individual bats and flight days. As we also predicted, shifts in the direction of beam aim were related to behavioral performance. In failed flights, bats largely failed to aim their beam in the direction of the turn; instead, they aimed their beam straight ahead, slightly toward the new turn, or toward the previously encountered opposite turn. The few calls aimed in the correct direction indicates that the bats probed both sides of the corridor even though calls are focused in one particular (in this case, incorrect) direction. These data support the hypothesis that it is the failure to switch beam aim that underlies deficits in behavioral performance.

Consistent with the formation of a spatial map (Barchi et al., 2013), bats adjusted their beam to the direction of the turns as they gained more experience with the spatial layout of the flight corridor. All bats initially failed to switch their beam aim when first introduced to 90° turns, and this was reflected in their poor performance. To increase performance, bats needed to adjust their beam aim to focus on the chains surrounding the turn. For the first day of flights in the new Right Turn condition, two bats continued to aim their beams straight ahead. Two other bats displayed more flexibility, shifting their calls in the direction of the new Right Turn on the first day they were exposed to it. By the end of the Right Turn condition, all bats shifted their beam aim to the correct side and all completed the task successfully. On the first day of Left Turn flights, all bats aimed their beam within ±9° of the midline. As the bats gained more experience in this condition, two of them adjusted their beam aim toward the left and successfully completed the task. Two other bats did not adjust their beam aim and failed the task (Figure 4). These data point to considerable individual variability in how flexible bats are when it comes to revising a learned spatial layout.

A strategy of focusing the center of the sonar beam on the nearest obstacle preventing a turn is an adaptive strategy that allows bats to localize and navigate tight spaces, such as gaps in foliage, more accurately. The center of the beam provides the highest signal-to-noise ratio (SNR) for echoes from any potential obstacle compared with the periphery (Ghose and Moss, 2003; Knowles et al., 2015). Focusing its calls on the nearest chain at the “corner” of the turn would allow the bat to perceive the first object to be avoided with a high SNR. However, aiming at the corner chain in the upcoming gap requires perceiving the open gap in the chains before actually reaching the turn, in spite of the intervening chains that prevent flight in the direction of the turn before physically arriving at the turn. For a bat flying at speed, the ability to perceive the two surfaces that define a gap accurately is crucial for localizing and perceiving the spatial properties of that gap, whether the surrounding obstacles are foliage or hanging chains. It is presumably for this reason that in successful turn flights bats focus on the area around the first chain defining the gap. This strategy of focusing the center of their beam on the upcoming obstacle reinforces previous results showing that big brown bats preferentially use the center of their beam to localize and track goal-related objects (Ghose and Moss, 2003; Surlykke et al., 2009), in contrast to other echolocating bats that employ an alternative spatial-sampling strategy to localize targets (Yovel et al., 2010, 2011). The overall conclusion is that the bats were able to perceive the upcoming open gap in the corridor and anticipate the upcoming turn both by slowing down and by aiming their broadcasts into the gap.

Changing beam aim based on an acquired spatial memory of the flightpath is beneficial to bats that must navigate tight spaces, allowing them to collect as much information as possible about upcoming obstacles in the direction of flight. However, an early proactive movement of the beam may make the bat less able to adapt to abrupt changes in the environment, even after multiple encounters with a new flightpath. Bats may incur a perceptual cost by shifting their acoustic gaze too early; a portion of their approach to the turn is spent echolocating off-axis from the area into which they will fly before entering the turn. Any echoes received from this immediately upcoming area would be perceived as clutter and perceptually blurred (Bates et al., 2011), making it difficult to perceive its distance and structure. The bats' built-up spatial memory of the flightpath allows them to incur this perceptual cost for the benefit of spending more time gathering perceptual information about the upcoming gap.

### Call timing is related to task difficulty

Insectivorous echolocating bats increase their sensory acquisition rate as background clutter becomes physically closer or denser, and as they approach a landing surface or wall (Falk et al., 2014; Lewanzik and Goerlitz, 2021; Moss et al., 2006; Petrites et al., 2009; Wheeler et al., 2016). The changes in call timing and IPI in our experiment reinforce these earlier findings. Mean IPIs were similar in successful Straight, Right Turn, and Left Turn flights. Bats increased IPIs as they flew down the Straight corridor and as they gained more experience with this flightpath, suggesting that this flight condition was perceptually easier than the Turn conditions. Bats decreased IPIs as they approached turns in the flightpath, indicating that these flights were more challenging. IPIs decreased more in successful Left Turn than Right Turn flights, highlighting the increased perceptual difficulty of these left turns. Over experimental days, IPIs increased in Straight and Right Turn flights but decreased in Left Turn flights. IPIs also decreased in failed flights. These shorter IPIs again indicate the perceptual difficulty of the Left Turn flights in particular.

We predicted that more calls would be emitted as SSGs in the more difficult Turn conditions, and the data support this prediction. These data are also consistent with previous work showing more call clustering in tasks presumed to be more perceptually difficult (Beetz et al., 2019; Lewanzik and Goerlitz, 2021; Moss et al., 2006; Petrites et al., 2009; Wheeler et al., 2016). SSG production helps the bats alleviate pulse-echo ambiguity created by multiple closely spaced rows of acoustic clutter (Accomando et al., 2018; Knowles et al., 2015; Petrites et al., 2009; Wheeler et al., 2016). In our experiment, the most common SSG was a doublet (mean 61%, range 42%–82% in successful flights in the three conditions). Other experiments in which bats flew down different-sized corridors surrounded by various densities of clutter reported doublets at 62% (Warnecke et al., 2016, 2018) and 37% (Wheeler et al., 2016) of all SSGs. In our experiment, an average of 15% (range 6%–22%) of SSGs were classified as triplets, similar to that found by Wheeler et al. (16%; 2016) but larger than identified in earlier work with FM bats (< 10%; Warnecke et al. [2016];, <5%, Beetz et al. [2019]). Doublets were the most common SSGs emitted in failed Left Turn flights. Two bats that were unable to perform these left turns produced more SSGs than in successful Right Turn flights. We interpret this finding to suggest that bats attempted to update their acoustic scene even under conditions when they did not adjust their beam aim to point toward the correct portion of the corridor.

The visualization of pre/post-IPI distributions for individual bats indicates that individual bats use unique patterns of call timing in order to solve the same perceptual task, and that these unique patterns within individuals are consistent across changes in the surrounding environment. Indeed, the pre-IPI to post-IPI distributions in failed flights were similar to those in successful flights by the same individual. Variability in task performance among bats is not attributable to changes in the temporal patterns of sensory acquisition, but rather due to individual differences in the spatial sampling strategies employed.

### General discussion

Together, these findings paint a picture of how bats modify their biosonar behaviors to perceive upcoming obstacles (chains), adjust their calling patterns to avoid these obstacles, and adapt their beam aim to drastic changes in the spatial environment. Bats localizing and navigating a gap within dense acoustic clutter focus their calls on the nearest obstacle defining the edges of the gap in order to best localize the relatively small gap. In effect, they make sonar “looks” into the surrounding clutter to identify upcoming openings amid multiple nearer objects and echoes arriving earlier than the absence of echoes signifying the opening. In this way, they can identify an open flightpath. These proactive beam aim changes are tightly integrated with a spatial memory of the bat's environment, which is built up with experience. This is best exemplified by the persistence of some bats in aiming their calls in the direction required by a previous condition, even after substantial experience in a new spatial layout. This spatial memory, required for successful navigation, may also inhibit the ability of bats to adapt to sudden changes in the environment. Bats that were able to unlearn a pattern of beam aim were more successful in navigating a novel, orthogonal flightpath compared with other bats that became “stuck” in a learned pattern of beam aim. These differences between individual bats could lead to significant differences in their ability to navigate and survive in volatile, physically changing environments. We conclude that failures in navigating complex flightpaths are due to the bat being unable to alter its beam aim, not to an inability to change its sensory acquisition rate. Final success reflects the bat's ability to form a new spatial map of its environment (Barchi et al., 2013).

### Limitations of the study

One issue raised by this experiment concerns the poor performance of two of the four bats in the Left Turn condition. It is possible that the Left Turn was more challenging because it was closer to the flight room wall. The simultaneous use of the flight room for other experiments prevented locating the experimental corridor in a more symmetrical manner. The better performance of these two bats in a Reversed Right Turn condition suggests that proximity to the wall did not affect their performance; rather, they did poorly in the Left Turn because they became “stuck” in aiming their beam toward the right. However, this new condition could only be run for one day, because of the bats' refusal to continue flying. These two bats were in good health during the experiment, and remained so for one year after its end. Finally, although the LMM model controlled statistically for the fixed order of conditions, future experiments should randomize conditions. It is also more difficult to quantify changes in temporal patterning that may have occurred in response to spatial changes, as time spent in the experiment is a confounding factor for all bats.

### Resource availability

#### Lead contact

Further information and requests should be directed to and will be fulfilled by the corresponding author, Andrea Simmons (Andrea_Simmons@brown.edu).

#### Materials availability

Please contact the corresponding author.

#### Data and code availability

Original data have been deposited in the Brown Digital Repository: https://doi.org/10.26300/0mpw-9f40.

## Methods

All methods can be found in the accompanying Transparent Methods supplemental file.

## Supplemental information

Document S1. Transparent methods, Figures S1–S3, and Tables S1 and S2
