# Supplementary material for: Spatiotemporal patterning of acoustic gaze in echolocating bats navigating gaps in clutter
Source: iScience. 2021 Mar 23;24(4):102353. doi: 10.1016/j.isci.2021.102353 (PMC8047172; doi:10.1016/j.isci.2021.102353)
Supplement: Document S1. Transparent methods, Figures S1–S3, and Tables S1 and S2 [file mmc1.pdf]

iScience, Volume *24*

Supplemental information

Spatiotemporal patterning of acoustic gaze in echolocating bats navigating gaps in clutter

Amaro Tuninetti, Chen Ming, Kelsey N. Hom, James A. Simmons, and Andrea Megela Simmons

Key Resources Table

REAGENT or RESOURCE	SOURCE	IDENTIFIER
Deposited data		
Raw and analyzed data	This paper	https://doi.org/10.26300/0mpw-9f40
Experimental models: organisms/strains		
Big brown bats, *Eptesicus fuscus*	Wild-caught	NA
Software and algorithms		
MATLAB	MathWorks, Natick MA	Version 2018a
SPSS	IBM Corp, Armonk NY	Version 25
R Studio	Rstudio.com	2020, Version 3.6.3
GraphPad	GraphPad Software, San Diego CA	Version 9.0.0
Other		
Electret microphones	Knowles Electronics, Itasca IL	SMG-0291
Data acquisition system	Astro-Med, West Warwick RI	DMX-8000

Transparent Methods

Animals

Animal care and experimental procedures were approved by the Brown University Institutional Animal Care and Use Committee and adhere to federal guidelines. Four adult big brown bats (three males, one female) were captured from barns in Rhode Island USA, authorized by a scientific collecting permit from the state of Rhode Island. Because these bats were wild-caught, their ages are not known. All bats flew and echolocated normally, as recorded in exercise flights conducted in an empty flight room. Animals were group-housed in a temperature- and humidity-controlled colony room (22-24° C, and 40-60% relative humidity) on a reverse 12:12 dark/light cycle. Individuals were identified by unique haircuts on their back fur. Bats had unlimited access to vitamin-enriched water in their home enclosures and received their daily food allotment (live mealworms, *Tenebrio* larvae) during experiments as rewards for successful performance. If they did not obtain the total food allotment during experiments because of poor performance, they were fed subsequently in their home enclosures. Two animals (Bat 1 and Bat 4) participated in previous experiments that involved flying through chain arrays of varying densities and configurations but lacking abrupt spatial changes (Hom et al., 2016; Simmons et al., 2018; Wheeler et al., 2016); two other animals (Bat 2 and Bat 3) were naïve to flight experiments.

Flight Room

Flights took place in a custom-built flight room (8.3 m × 4.3 m × 2.7 m; Figure 1) lined with acoustically and electrically insulated sound-absorbent foam (SONEX "One" acoustic panels; Pinta Acoustics, Minneapolis, MN USA) on the ceiling and walls. The floor was lined with carpet to help attenuate echoes. Black plastic chains (217 total; link size 4.0 cm wide, 7.5 cm long, 1.0 cm thick) were suspended from cross bars in the ceiling and extended to the floor to create a straight, 5 m long, 40 cm wide corridor. These chains reflect intense echoes (87-97 dB SPL re 20 µPa as measured by Petrites et al., 2009) that are extended in time, and are analogous to echoes reflected by dense vegetation. Chains were arranged in rows and columns 20 cm apart and were weighted on the floor end to keep them from swaying. This is a denser arrangement than in earlier experiments (Hom et al., 2016; Petrites et al., 2009; Wheeler et al., 2016) and was chosen in order to prevent bats from easily flying out of the experimental flightpath when it was abruptly changed. In the plan diagram of the flight room (Figure 1), filled circles show the positions of hanging chains and open circles show the position of chains removed to construct different corridor configurations (Straight, 90° Left Turn, 90° Right Turn, Reversed Right Turn). The chain array is not located symmetrically in the flight room because it was constrained by equipment needed for unrelated experiments. When the bat turned to the right, it had a farther distance to fly to Wall C then when it turned left towards Wall A. This lack of symmetry led to the inclusion of a Reversed Right Turn condition.

Two thermal video cameras (v1 and v2) and 16 ultrasonic microphones (numbered boxes, Figure 1) were used to monitor the bat during the experiment. The thermal infrared cameras (Merlin mid-range Photon 320, FLIR Systems, Boston, MA USA) were placed at the beginning of the flight corridor aiming down its length, and in the middle of the corridor facing the floor. Thermal video feeds were used for live monitoring of the bat's flight. Fifteen ultrasonic microphones (SMG-0291 electret microphones, Knowles Electronics, Itasca, IL USA) were placed on Walls A, B, and C, and one (#16) on the ceiling above the turning point (or the corresponding point in Straight flights), to create a microphone array capturing the bat's echolocation calls as it flew. The wall microphones were mounted 0.5, 1.2, 1.6, or 2.1 m above the ground, in an alternating high-low order. This array was used to calculate the bat's position within the corridor at the time of emission of each individual call as well as the direction in which the call was emitted.

To ensure that the bats were not relying on any visual cues to navigate the corridor, flights took place in full darkness, though the room was illuminated by infrared LEDs mounted on the walls for video recording and monitoring purposes.

Supplemental Experimental Procedure

Flights were conducted by three experimenters. Experimenter 1 was responsible for releasing the bats at the start of the corridor, retrieving them when they landed on the wall, and then rewarding them for successful flights. Experimenter 2 turned on the acoustic recording equipment prior to the bat's release

and stopped the equipment when the bat landed on the back wall, while Experimenter 3 took notes on each flight.

Bats were exposed to different corridor configurations (flight conditions) in a fixed order. The experiment began with 7 days of flights through the Straight corridor. All bats then completed 9 days of flights in the Right Turn condition (towards Wall C) and finally 6-7 days of flights in the Left Turn condition (towards Wall B). Two bats were tested for one day in a Reversed Right Turn condition, being released from Wall B to find the gap leading towards Wall A. Each bat completed 15-18 flights per day, with the goal of 15 successful flights.

Data analysis

Performance

The data collected from each flight consisted of ultrasonic recordings of the bat's echolocation calls and its performance (success or failure). Any flight in which the bat successfully flew from the beginning of the corridor to the appropriate wall without looping back to the release point, landing on a chain, exiting the corridor through the chains, or falling to the floor was labeled a success and rewarded with a mealworm. Any flight where the bat landed on the floor or on a chain, looped back to the release point without completing the flightpath, flew through the chains, or refused to fly was labelled a failure. Bats were not rewarded for failed flights.

Calculation of flightpath, beam aim, and flight speed

Recordings from all 16 microphones were digitized in real time at a 192 kHz sampling rate using a commercial multichannel data-acquisition system (DMX-8000, Astro-Med Inc., West Warwick, RI USA) and archived in the recording system's dedicated computer. For off-line analysis, they were recovered from the archived format, converted to mono .wav files, and bandpass filtered from 15-75 kHz to remove background noise unrelated to the bat's calls and exploit the strongest part of the signals relative to background noise in the room. This frequency band was used to estimate arrival-time differences at the microphones. Calls recorded from multiple microphones started on the same clock at known positions were cross-correlated to determine the time-difference-of-arrival between channels and the locus of the source for each individual call (Barchi et al., 2013; Gillette and Silverman, 2008; Wei and Ye, 2008). For an individual call to be analyzed, we required that the call be successfully identified on the recordings of seven wall microphones. This threshold was chosen to provide an accurate reconstruction of the location and beam aim of each call while also maximizing the number of calls emitted that could be used for beam aim analysis. The number of channels used to localize a call ranged from 7 to 16, depending on the emitted strength and recording quality of each call.

Beam aim and flightpaths were calculated using custom MATLAB scripts (R2018a, MathWorks, Cambridge, MA USA). For cross-correlation, a single call from each flight was chosen as a reference call. Selecting an intact and strong representation of a bat's call as reference is critical to the accuracy of tracking. Most of the calls emitted within a flight encountered several rows of chains before being recorded by a wall microphone, which results in recordings that can be degraded and contain many spectral notches. However, the directly received call was stronger than the scattered sound, which, being weaker, did not affect the principal peak of the cross-correlation. The microphones nearest the entrance of the corridor (#14 and #15) were able to record initial calls at relatively high amplitudes that were not highly scattered. In some flights, calls reaching these microphones were overly scattered or captured off-axis from the bat, resulting in recordings of calls missing the second harmonic. In these cases, a recorded call from the ceiling microphone (#16) was used as the reference call for that flight, as calls reaching that microphone were not highly scattered by chains.

Once individual calls were localized within the flightpath, the horizontal direction (azimuth) of each call's broadcast beam was calculated. While localization was performed by time-of-arrival measurements using the full band of the FM calls (15-75 kHz), with no role for amplitude or intensity estimates affecting the time values, determining beam aim depends on locating the direction of maximum amplitude using signals recorded by multiple microphones. To mitigate the potential for distortion of beam aim estimates caused by the scattering effect of the chains on any particular microphone's estimate of call amplitude, we bandpass filtered (equiripple FIR filter) the calls to 30-32 kHz. This frequency band is strongly

represented in big brown bat echolocation calls, and these lower frequencies travel more effectively than the higher frequencies in the full call bandwidth without being scattered by the 1.0 cm thick loops on the chains. The intensity of a call at each microphone was obtained by squaring the bandpass filtered signal to obtain its envelope and then integrating this squared envelope to arrive at a single intensity value for that call. After correcting the signal intensity at each microphone for spherical loss and atmospheric attenuation incurred over the path length from the location of the call, the beam axis of each call was calculated by summing all direction vectors whose lengths are proportional to the corrected intensity to find the central axis of the beam (Ghose and Moss, 2003). A total of 14 microphones was used in the beam aim calculation. Microphone #10 was excluded because in a large number of flights the recording from this microphone appeared abnormal, likely due to a bad connection at the time of experiments; microphone #16 was placed centrally on the ceiling and thus did not provide azimuthal information.

To quantify trends in beam aim during each flight, we averaged beam angle across overlapping windows of the flight corridor. The section of corridor preceding the 90˚ turn was divided into 10 cm windows. The mean and SD of beam aim angle was calculated for all pulses emitted within that window. Mean beam aim was determined to deviate from the midline in turn conditions when the SD of beam aim angles across all emitted calls no longer overlapped the 0˚ line (facing straight ahead in the chain array).

Flight speeds on successful flights were calculated from the pulse timing and localization data using custom MATLAB scripts. Speed in each of two segments of the corridor, as referred to the position of the turn at 0 cm (speed 1, 250 – 100 cm; speed 2, 100 – 0 cm; orange and purples lines, respectively, in Figure 1), was computed by selecting the calls that most closely corresponded to the Y-positions of the selected ranges, and calculating the corresponding time-lapse between selected calls. The nearer segment (100 – 0 cm before the turn) was chosen because individual bats diverted their beam angle away from the midline at various points between 100 – 0 cm from the turn (see Figure 4), meaning the bats began physically preparing for the turn within 100 cm from the turn. The farther segment (250 – 100 cm) was chosen as the comparison range because this segment was most representative of the bat's stable flight speed in the straight section of the turn conditions. Distances farther than 250 cm from the turn were excluded to avoid variable flight speeds due to the bat's initial release. Failed flights were excluded. Speeds greater than 5 m/s most likely are errors caused by reflections from the chains and were also excluded. No videos showed bats flying the array length in under 1 s, which would happen at such high speeds. We compared speed distributions in the two corridor segments for each individual bat using two-tailed Kolmogorov-Smirnov tests.

Temporal pattern of call emissions

We calculated numbers of calls and time intervals between individual calls (interpulse intervals, IPI) from the time the bat entered the corridor to the time it reached the turn or equivalent point in Straight flights. An amplitude threshold was set in MATLAB to isolate individual pulses from background noise and to calculate the time-of-occurrence of each pulse. Because different bats emitted calls at different energy levels, the amplitude threshold differed between bats but was kept the same to analyze individual flights from the same bat. To avoid including echoes rather than emitted pulses in the analysis, a minimum time of 10 ms between individual pulses was set. In addition, IPIs > 100 ms were excluded, as these indicate that the bat is not in the corridor. The time between amplitude maxima of pulses was calculated to determine IPI. Sonar sound groups (SSGs) were classified as singles, doublets, triplets, quadruplets, and more based on differences in IPI within groups and between groups of pulses. Classification was done by analyzing IPI values with the algorithm presented in Kothari et al. (2014), with modified criteria (stability criterion = 8%, island criterion = 1.1; Warnecke et al., 2016, 2018) developed to account for greater densities of acoustic scenes. The algorithm was run without a maximum SSG size, so as to let the algorithm classify SSGs of any size based on algorithm criteria. SSG classifications were visually confirmed to ensure algorithm criteria did not produce false positives. The temporal patterning of calls was visualized by plotting the distribution of calls that were classified as SSGs, plotting the results of linear mixed effects models showing how IPI changed over flight time, and plotting pre-IPI against post-IPI for calls emitted in flight (Wheeler et al., 2016).

Statistical analyses

Performance data were analyzed by repeated measures analysis of variance (ANOVA; SPSS v. 25), with bat as the random factor and condition (Straight, Right Turn, Left Turn) as the fixed factor. The distance at which beam aims shifted in the direction of a turn was determined by when the SD of beam aims in a window no longer overlapped the flight path's midline (0˚). Beam shift linear regressions from the first and last day of flights were compared with ANCOVA pairwise comparisons (Bonferroni-corrected $\alpha = 0.004$; GraphPad Software v9.0.0).

We analyzed IPIs using linear mixed effects models (LMM) using the *lmer4* package in RStudio (2020, R version 3.6.3). Mixed effects models account for the hierarchical repeated-measures structure of the data (each individual bat emits *n* calls, resulting in *n*-1 IPIs, on flight *x*, on day *y*, of condition *z*) as well as datasets of unequal *n* without discarding data. The variables Bat, Flight Number, Day Number, and Condition were included as nested random effects. The hierarchical random effects structure of the first LMM helps account for the fact that all bats completed the different flight conditions in the same, as opposed to randomized, order (Straight, Right Turn, Left Turn) and for several consecutive days each. In this LMM, three fixed effects were included: Condition, the Number of Calls remaining before reaching the turn, and the interaction between those two effects. The number of calls remaining before the bat reached the turn was used to estimate how close the bat was to entering the turn (or the equivalent point in Straight flights). Precise physical distance from the turn is not known for every single call, as precise localization of the bat required a call to be picked up by seven microphones. Thus, we identified the final call that was successfully localized as being emitted before the entrance to the turn, and indexed the number of calls remaining (before reaching the turn) backwards from the final call. To test whether changes in IPI while approaching the turn were different across the three conditions, pairwise comparisons of the condition*calls-to-turn interaction were performed by contrast coding in order to compare IPI slopes in the Right and Left Turn flights to the slope in Straight flights (Wendorf, 2004). Significance of effects and pairwise comparisons was determined using the *lmerTest* package in RStudio, which performs F tests using Satterthwaite's degrees of freedom method (Brown, 2020).

We fit IPI data to a second LMM quantifying how IPI changed over the course of 7-9 days of flying in the same configuration of chains, as each bat flew within a single configuration for 7-9 continuous days before switching to another chain configuration. This LMM differs in two ways from the previous IPI model. First, it replaces the fixed effect of Number of Calls to turn with a fixed effect of Number of Days, which quantifies how many consecutive days the bat has been flying in a condition. Second, the fixed effect interaction is now between Condition and Number of Days (rather than Number of Calls).

To test for changes in SSGs, we used McNemar repeated-measures chi-squared tests (SPSS, v. 25) to determine if, when introduced to a new task condition, bats significantly changed the proportion of calls they emitted as single calls and the proportion of calls they emitted as parts of an SSG.

Supplemental References

Barchi, J. R., Knowles, J. M., and Simmons, J. A. (2013). Spatial memory and stereotypy of flight paths by big brown bats in cluttered surroundings. J. Exp. Biol. *216*(6), 1053–1063. https://doi.org/10.1242/jeb.073197

Brown, V. A. (2020, April 11). An introduction to linear mixed effects modeling in R. https://doi.org/10.31234/osf.io/9vghm

Ghose, K. and Moss, C. F. (2003). The sonar beam pattern of a flying bat as it tracks tethered insects. J. Acoust. Soc. Am. *114*(2), 1120–1131. https://doi.org/10.1121/1.1589754

Gillette, M. D. and Silverman, H. F. (2008). A linear closed-form algorithm for source localization from time-differences of arrival. IEEE Signal Process. Lett. *15*, 1–4. https://doi.org/10.1109/LSP.2007.910324

Hom, K. N., Linnenschmidt, M., Simmons, J. A., and Simmons, A. M. (2016). Echolocation behavior in big brown bats is not impaired after intense broadband noise exposures. J. Exp. Biol. *219*, 3253-3260.

https://doi.org/10.1242/jeb.143578

Kothari, N. B., Wohlgemuth, M. J., Hulgard, K., Surlykke, A., and Moss, C. F. (2014). Timing matters: Sonar call groups facilitate target localization in bats. Front. Physiol. *5*, 1–13. https://doi.org/10.3389/fphys.2014.00168

Petrites, A. E., Eng, O. S., Mowlds, D. S., Simmons, J. A., and Delong, C. M. (2009). Interpulse interval modulation by echolocating big brown bats (*Eptesicus fuscus*) in different densities of obstacle clutter. J. Comp. Physiol. A *195*(6), 603–617. https://doi.org/10.1007/s00359-009-0435-6

Simmons, A. M., Ertman, A., Hom, K. N., and Simmons, J. A. (2018). Big brown bats (*Eptesicus fuscus*) successfully navigate through clutter after exposure to intense band-limited sound. Sci. Rep. *8*, 13555. DOI:10.1038/s41598-018-31872-x

Warnecke, M., Lee, W-J., Krishnan, A., and Moss, C. F. (2016). Dynamic echo information guides flight in the big brown bat. Front. Behav. Neurosci. *10*, 81. doi: 10.3389/fnbeh.2016.00081

Warnecke, M., Macías, S., Falk, B., and Moss, C. F. (2018). Echo interval and not echo intensity drives bat flight behavior in structured corridors. J. Exp. Biol. *221*, jeb191155, 2018. doi:10.1242/jeb.191155

Wei, H.-W., and Ye, S.-F. (2008). Comments on ‘A linear closed-form algorithm for source localization from time-differences of arrival’. IEEE Signal Processing Letters *15*, 895–895. https://doi.org/10.1109/LSP.2008.2001113

Wendorf, C. A. W. (2004). Primer on multiple regression coding: Common forms and the additional case of repeated contrasts. Understanding Statistics, *3*(1), 47-57.

Wheeler, A. R., Fulton, K. A., Gaudette, J. E., Simmons, R. A., Matsuo, I., and Simmons, J. A. (2016). Echolocating big brown bats, *Eptesicus fuscus*, modulate pulse intervals to overcome range ambiguity in cluttered surroundings. Front. Behav. Neurosci. *10*, 1–13. https://doi.org/10.3389/fnbeh.2016.00125

Figure S1. **Beam angles in failed flights.** Related to Figure 4.
Beam aim is calculated in the segment of the flight path from 150 cm – 0 cm to the turn (or equivalent point in Straight flights; beam 2 in Figure 1). Empty cells indicate no data for that condition. In failed Left Turn flights, Bat 3 and Bat 4 continued to (mistakenly) aim their beams towards the right as they approached the turn.

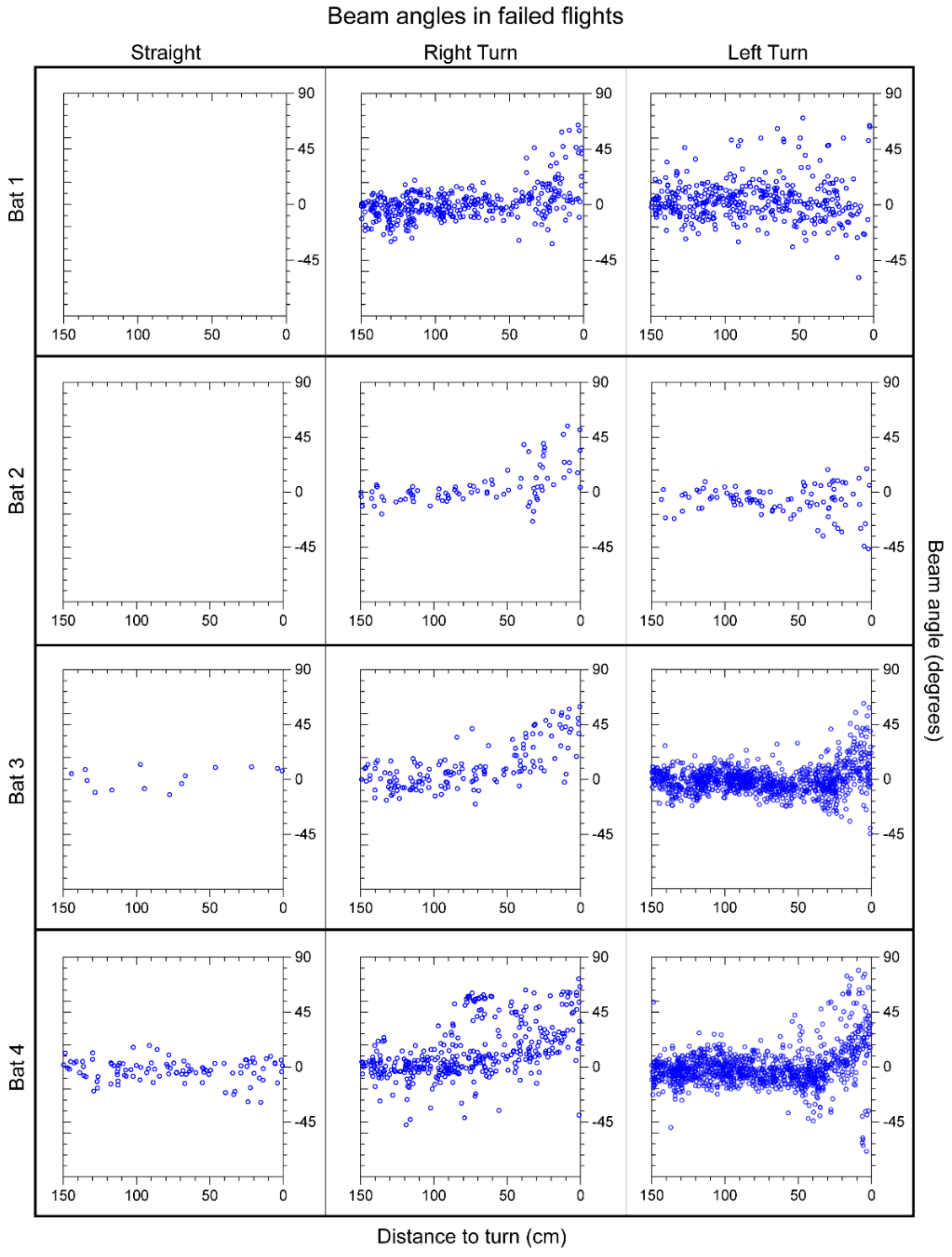

Figure S2. Flight speeds in successful flights. Related to Figure 1 and Figure 4.
Speeds were calculated separately in two segments of the corridor (Figure 1): 250 – 100 cm prior to the turn (speed 1, orange bars), and 100 – 0 cm prior to the turn or equivalent point in straight flights (speed 2, purple bars). Columns (left to right) show data for Straight, Right Turn, and Left Turn flights. Flight speeds in failed flights were not calculated.

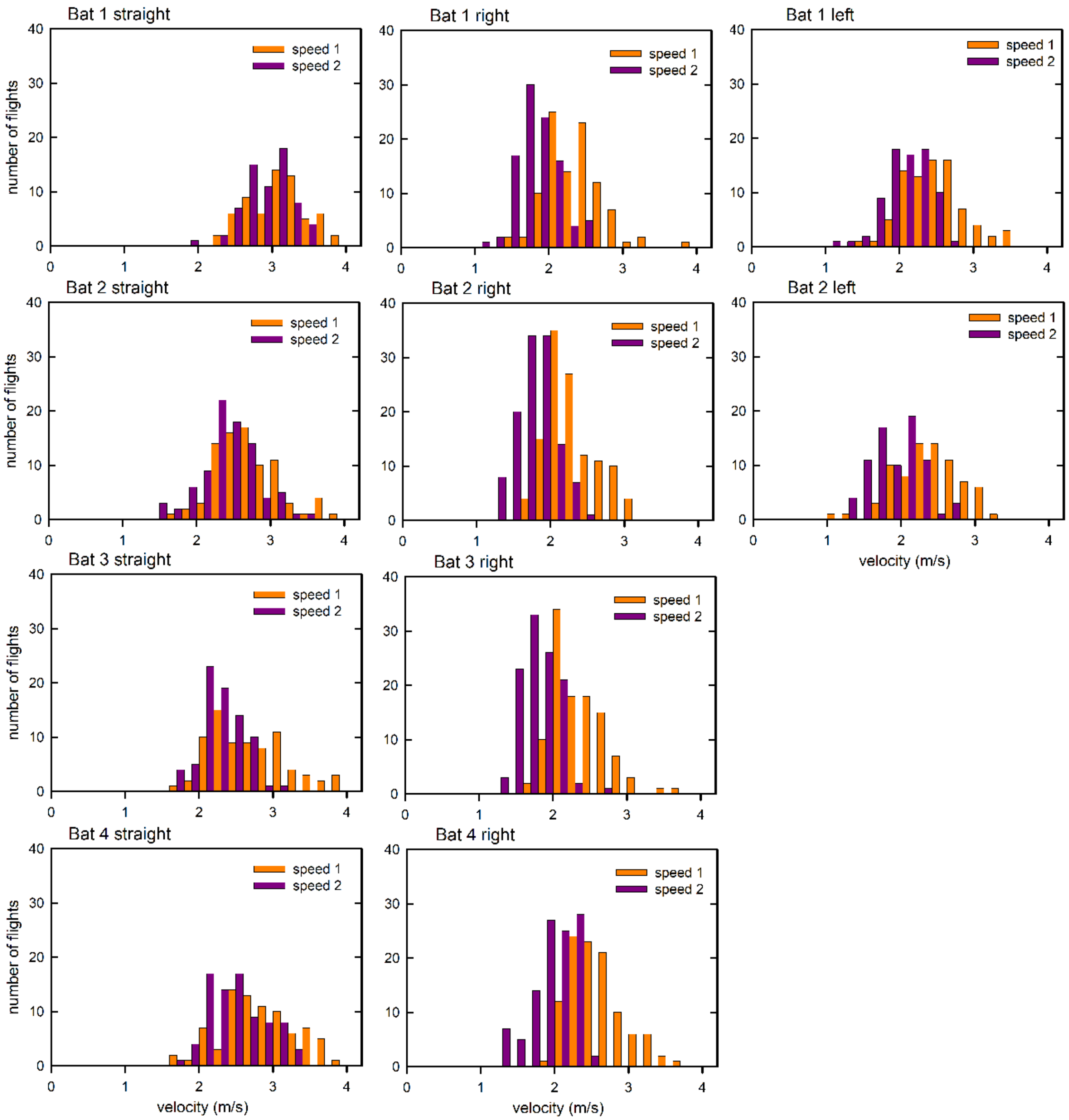

Figure S3. **Spectrograms of calls during a successful (top panel) and a failed (bottom panel) flight through the Right Turn corridor.** Related to Figure 6.
Data are from Bat 1. White labels and numbers identify SSG categorizations and the number of pulses within each group. White triangles with distance measurements indicate the bat's position relative to the upcoming turn (beam 2 segment in Figure 1). In the failed Right Turn flight, the bat failed by colliding with the chains blocking the entrance to the turn (note the buzz-like call structure leading up to the turn).

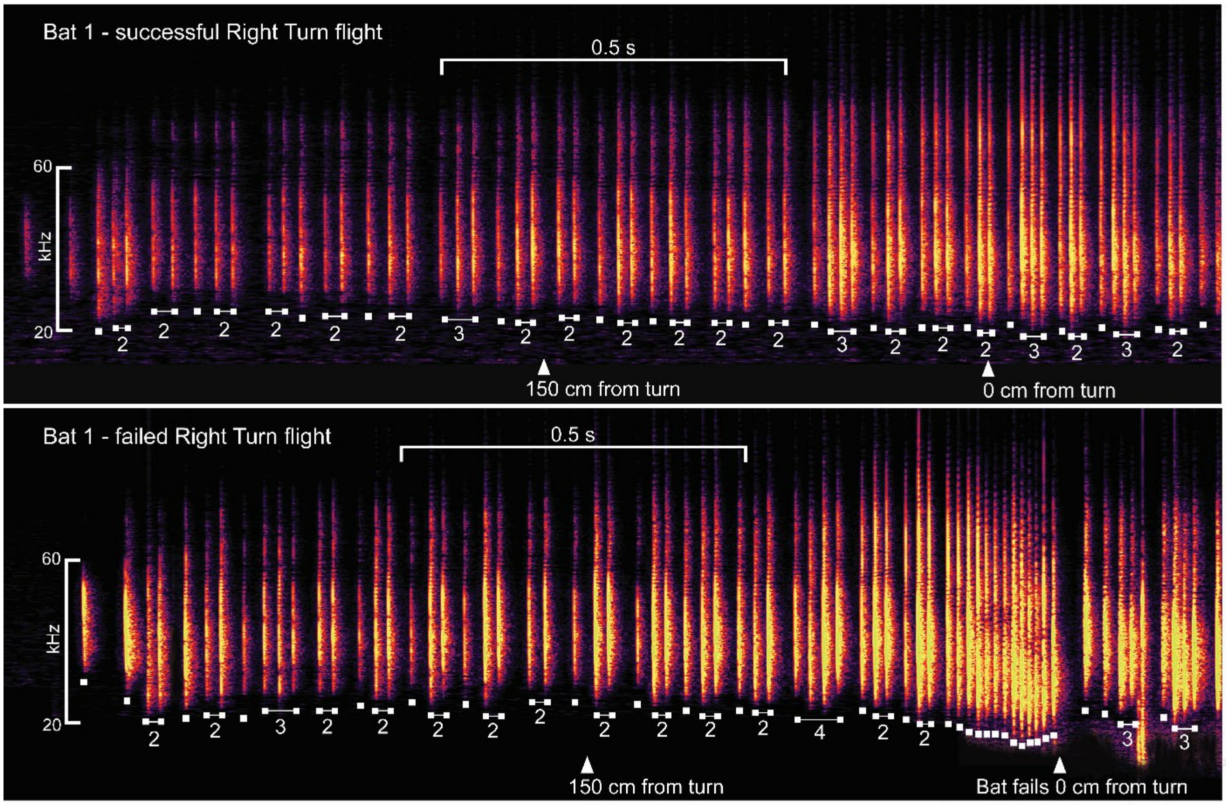

Table S1. **Mean beam angles of calls on failed flights**. Related to Table 1.
Beam angles are calculated for two segments of the corridor (beam 1, 300 – 150 cm; beam 2, 150 – 0 cm; see Figure 1), referenced to distance to the turn. N = number of angles (followed by the number of flights in parentheses), M = mean, SD = standard deviation. Empty cells indicate no data from failed flights for that combination of bat and condition. Italicized values highlight those from the two bats that were experienced in flight tasks.

**Condition**	**Distance to turn**		***Bat 1***	**Bat 2**	**Bat 3**	***Bat 4***
**Straight**	300 – 150 cm	**N [calls (flights)]**		11 (2)	14 (4)	*150 (8)*
		**M**		4.69°	8.91°	*2.27°*
		**SD**		12.75°	19.83°	*10.95°*
	150 – 0 cm	**N [calls (flights)]**			14 (2)	*47 (5)*
		**M**			1.92°	*4.98°*
		**SD**			8.64°	*4.16°*
**Right Turn**	300 – 150 cm	**N [calls (flights)]**	*622 (27)*	78 (8)	202 (20)	*487 (22)*
		**M**	*-0.98°*	-1.16°	3.37°	*3.61°*
		**SD**	*9.06°*	6.47°	11.22°	*8.57°*
	150 – 0 cm	**N [calls (flights)]**	*220 (27)*	66 (8)	117 (20)	*280 (20)*
		**M**	*16.39°*	22.22°	25.41°	*23.42°*
		**SD**	*20.43°*	19.87°	22.26°	*20.87°*
**Left Turn**	300 – 150 cm	**N [calls (flights)]**	*505 (20)*	46 (8)	1011 (80)	*1351 (72)*
		**M**	*4.80°*	-2.80°	0.90°	*-0.94°*
		**SD**	*10.64°*	6.83°	7.89°	*9.57°*
	150 – 0 cm	**N [calls (flights)]**	*238 (20)*	38 (8)	539 (76)	*1221 (67)*
		**M**	*14.17°*	10.59°	12.71°	*25.02°*
		**SD**	*14.97°*	12.19°	14.87°	*18.18°*

Table S2. Proportions of all calls categorized as SSGs of varying size. Related to Figure 7. SSG size (left column) varies from 1 (single calls) to 8 (octuplets). SSGs were classified using the algorithm used by Warnecke et al. (2016, 2018). The values from Bat 3 and Bat 4 in the Left Turn condition (italicized columns) are based on failed flights only; all other values are based on successful flights only. Proportions of single calls/SSGs in the Turn conditions are significantly different from proportions in the Straight condition (McNemar repeated-measures chi-square tests, all P values < 0.001).

	**Bat 1**			**Bat 2**			**Bat 3**			**Bat 4**		
**SSG size**	Straight	Right Turn*	Left Turn*	Straight	Right Turn*	Left Turn*	Straight	Right Turn*	*Left Turn**	Straight	Right Turn*	*Left Turn**
**1**	0.224	0.242	0.261	0.131	0.078	0.288	0.201	0.200	*0.218*	0.284	0.293	*0.271*
**2**	0.448	0.573	0.474	0.672	0.817	0.627	0.708	0.735	*0.629*	0.478	0.541	*0.393*
**3**	0.284	0.165	0.225	0.183	0.100	0.067	0.087	0.056	*0.121*	0.217	0.154	*0.277*
**4**	0.040	0.017	0.036	0.009	0.005	0.008	0.004	0.007	*0.027*	0.017	0.012	*0.041*
**5**	0.003	0.003	0.002	0.004	0.000	0.003	0.000	0.001	*0.004*	0.002	0.001	*0.009*
**6**	0.000	0.000	0.002	0.000	0.000	0.003	0.000	0.000	*0.000*	0.001	0.000	*0.004*
**7**	0.002	0.000	0.000	0.000	0.000	0.000	0.000	0.000	*0.000*	0.000	0.000	*0.004*
**8**	0.000	0.000	0.000	0.000	0.000	0.004	0.000	0.000	*0.000*	0.000	0.000	*0.000*
